# Bone marrow stromal cells promote chemoresistance in acute myeloid leukaemia through P2RX7-mediated antioxidant defence

**DOI:** 10.1038/s12276-026-01839-4

**Published:** 2026-09-03

**Authors:** Ruolan You, Xiaoting Wang, Xiaoming Zheng, Wenqi Fang, Cen Jiang, Jingru Liu, Xiaolin Xu, Maoqing Tan, Danni Cai, Diyu Hou, Jing Zheng, Xinjian Lin, Huifang Huang

**Affiliations:** 1 Central Laboratory, Fujian Medical University Union Hospital, Fuzhou, Fujian, 350001 China; 2 Liangzhu Laboratory, Zhejiang University, Hangzhou, Zhejiang 311121 China; 3 Department of Clinical Laboratory, Wuhan Fourth Hospital, Wuhan, Hubei 430033 China; 4 Medical Technology and Engineering College of Fujian Medical University, Fuzhou, Fujian, 350122 China; 5 Fujian Institute of Hematology, Fujian Provincial Key Laboratory on Hematology, Fujian Medical University Union Hospital, Fuzhou, Fujian, 350001 China; 6 Key Laboratory of Gastrointestinal Cancer (Fujian Medical University), Ministry of Education, Fuzhou, Fujian, 350122 China

**Keywords:** Cancer microenvironment, Mechanisms of disease, Cell signalling

## Abstract

Acute myeloid leukaemia (AML) remains a therapeutically challenging malignancy owing to its high relapse rates and chemoresistance, often linked to elevated antioxidant defences. Here, we demonstrate that bone marrow stromal cells (BMSCs) confer chemoresistance in AML by upregulating the purinergic receptor P2RX7. Blocking P2RX7 restored chemosensitivity of AML cells. Mechanistically, BMSC-mediated P2RX7 overexpression induces calcium influx and mitochondrial calcium overload, triggering mitochondrial stress and a compensatory antioxidant response. This process involves mitochondrial phosphoglycerate mutase 5 (PGAM5) transducing reactive oxygen species signals, interfering with Keap1–Nrf2 complex formation and leading to Nrf2 stabilization, thereby enhancing cellular antioxidant defences. These findings identify a vital role of P2RX7 in the BMSC-driven redox adaptation programme that underlies AML chemoresistance and highlight P2RX7 signalling as a potential therapeutic target.

## P2RX7 Signalling Drives Chemoresistance in Acute Myeloid Leukaemia

Acute myeloid leukaemia (AML) is a complex blood cancer with poor survival rates, often due to drug resistance. This study explores how bone marrow stromal cells contribute to this resistance by enhancing antioxidant defences in AML cells. Researchers found that bone marrow stromal cells upregulate the purinergic receptor P2RX7 in AML cells, which increases mitochondrial calcium and reactive oxygen species levels. This mitochondrial stress activates the Nrf2 antioxidant pathway, enhancing cell survival against chemotherapy. The study used co-culture systems, genetic modifications and pharmacological interventions to demonstrate that P2RX7-driven mitochondrial stress stabilizes Nrf2, reducing its degradation and promoting chemoresistance. In in vivo experiments, blocking P2RX7 with AZ10606120 sensitizes AML cells to chemotherapy, reducing leukaemic burden. These findings suggest that targeting P2RX7 could improve AML treatment outcomes by overcoming microenvironment-induced chemoresistance, offering a promising therapeutic strategy.

This summary was initially drafted using artificial intelligence, then revised and fact-checked by the author.

## Introduction

Acute myeloid leukaemia (AML) is a highly heterogeneous haematological malignancy characterized by clonal expansion of immature myeloid cells and poor long-term survival. Although many patients achieve complete remission following standard induction chemotherapy with cytarabine and anthracyclines, the majority ultimately relapse owing to acquired drug resistance^1,2^. Mounting evidence suggests that the bone marrow microenvironment (BMME), particularly bone marrow stromal cells (BMSCs), plays a pivotal role in protecting AML cells from chemotherapy-induced cytotoxicity, thereby promoting disease persistence and relapse^3^. Understanding the molecular mechanisms by which the BMME confers this survival advantage is crucial for improving therapeutic outcomes.

AML cells exhibit a strong reliance on mitochondrial oxidative phosphorylation (OXPHOS) to meet their high bioenergetic and biosynthetic demands. BMSCs have been shown to support mitochondrial metabolism in AML cells, a feature closely associated with chemoresistance^4,5^. However, elevated mitochondrial respiration is intrinsically linked to increased production of reactive oxygen species (ROS), particularly within the mitochondria (mtROS)^6^. Although excessive mtROS can induce mitochondrial dysfunction and apoptosis, a moderate increase in mtROS can paradoxically trigger adaptive antioxidant responses that enhance cell survival — a process termed mitohormesis^7^. This adaptive mechanism enables AML cells to withstand oxidative damage and maintain redox balance under therapeutic stress. Recent studies have identified enhanced antioxidant capacity as a key contributor to drug resistance in multiple cancer types, including AML^8^. In particular, BMSCs have been implicated in promoting resistance to cytarabine by enhancing the antioxidant defences of AML cells^9,10^. However, the upstream signals and molecular mediators through which BMSCs induce these redox adaptations remain poorly defined.

Our previous work showed that mitochondrial ATP production was significantly elevated in AML cells co-cultured with BMSCs^4,11^. Because extracellular ATP is a key signalling molecule within the BMME, we turned on purinergic receptors as candidate mediators linking BMSC-derived metabolic cues to AML cell signalling. These receptors function as sensors of extracellular nucleotides and have been implicated in regulating tumour cell survival, metabolism and therapy response. The plasma membrane‑localized-purinergic receptor P2RX7, a ligand-gated ion channel activated by extracellular ATP, mediates calcium influx, potassium efflux, and downstream inflammatory and metabolic signalling^12^. In the context of AML, P2RX7 is found to be highly expressed in leukaemic blasts and leukaemia-initiating cells, and its elevated expression correlates with poor clinical outcomes^13,14^. Functionally, P2RX7 has been implicated in promoting leukaemic cell survival, proliferation and homing to the bone marrow niche^13,14^. Intriguingly, recent studies suggest that P2RX7 activation may also influence redox balance by regulating intracellular calcium dynamics and mitochondrial function, which are tightly linked to oxidative stress responses^15,16^. As a ligand-gated cation channel on the cell membrane, P2RX7 activation promotes Ca^2+^ and Na^+^ influx as well as K^+^ efflux^12^. This ion flux can increase mitochondrial Ca^2+^ (mtCa^2+^) accumulation and thereby drive mitochondrial oxidative stress^16^. However, whether this mechanism operates in AML and contributes to chemoresistance has not been fully defined. Therefore, investigating whether and how P2RX7 regulates AML chemoresistance through mitochondrial redox and antioxidant defence pathways is both biologically important and clinically relevant.

Nuclear factor E2-related factor 2 (Nrf2) is a master antioxidant transcription factor. Under basal conditions, Nrf2 is bound by the chaperone molecule Kelch-like ECH-related protein 1 (Keap1), which promotes its continuous ubiquitin-proteasomal degradation and thereby maintains redox homeostasis. Upon oxidative stress, Nrf2 escapes Keap1-mediated degradation, resulting in its stabilization, accumulation and nuclear translocation. Nuclear Nrf2 then binds to antioxidant response elements (AREs) in the promoter regions of a broad set of antioxidant and cytoprotective genes, including CAT, NQO1, HMOX1, thereby inducing a robust antioxidant-defence programme^17^. Although the molecular basis and biological significance of the Keap1–Nrf2 stress-sensing system have been extensively studied, the upstream mechanisms linking mtROS to Nrf2/ARE activation remain incompletely understood. Because disruption of the Keap1–Nrf2 complex and stabilization of Nrf2 are central to activation of this pathway, defining how mtROS regulates Keap1–Nrf2 signalling may provide important insight into antioxidant adaptation and chemoresistance in AML.

In parallel, phosphoglycerate mutase 5 (PGAM5) localized to the outer mitochondrial membrane, serves as a key sensor and effector of mitochondrial stress^18–20^. PGAM5 has been shown to modulate mitochondrial quality control and apoptosis^21^. Under stress conditions, PGAM5 may facilitate signal transduction from the mitochondria to the nucleus, influencing the transcriptional activation of antioxidant genes^22^. Nonetheless, it remains unclear whether PGAM5 mediates mitochondrial-to-nuclear communication in response to BMSC-induced oxidative stress in AML cells, and whether this process is essential for the activation of Nrf2-driven antioxidant programmes. Clarifying this signalling axis may uncover critical mechanisms by which the BMME supports leukaemic cell survival and resistance.

As a highly heterogeneous disease, AML develops drug resistance through multiple mechanisms, including drug efflux, enhanced DNA-damage repair, leukaemia stem-cell quiescence, epigenetic reprogramming, altered apoptotic priming, oncogenic kinase signalling and microenvironment-derived survival cues. In this study, we demonstrate that BMSCs act as microenvironmental inducers of P2RX7 upregulation in AML cells, thereby enhancing the antioxidant capacity and chemoresistance. Mechanistically, P2RX7 promotes mtCa^2+^ overload and mtROS production. This mitochondrial stress signal is sensed by PGAM5, which disrupts Keap1–Nrf2 complex formation, stabilizes Nrf2, and activates the Nrf2/ARE (antioxidant response elements) pathway. Our findings uncover a BMSC-driven mitochondrial-nuclear communication axis that promotes redox adaptation and resistance in AML, and identify P2RX7 signalling as a potentially targetable pathway that may be combined with chemotherapy to overcome microenvironment-induced chemoresistance.

## Materials and methods

Details on key resource are shown in Table 1.

**Table 1:** Material information.

Antibodies		
	Manufacturer	Catalogue number
Rabbit polyclonal anti-P2RX7 antibody	Sigma-Aldrich	HPA034967; RRID: AB_10601043
Mouse monoclonal anti-beta actin antibody (clone 2D4H5)	Proteintech	66009-1-Ig; RRID: AB_2687938
Rabbit monoclonal anti-Nrf2 antibody (clone EP1808Y)	abcam	ab62352; RRID: AB_944418
Rabbit anti-Nrf2 antibody (clone 1I21)	Proteintech	80593-1-RR; RRID: AB_2918904
Mouse monoclonal anti-Keap1 antibody (clone 4G10H9)	Proteintech	60027-1-Ig; RRID: AB_2132623
Rabbit polyclonal anti-Keap1 antibody	Proteintech	10503-2-AP; RRID: AB_2132625
Mouse monoclonal anti-PGAM5 antibody (clone CL0624)	abcam	ab244218
Rabbit monoclonal anti-Ubiquitin antibody (clone E4I2J)	Cell Signaling Technology	43124; RRID: AB_2799235
Rabbit monoclonal anti-IgG antibody (clone DA1E)	Cell Signaling Technology	3900; RRID: AB_1550038
Anti-rabbit IgG H&L antibody	Beyotime, China	A0208; RRID: AB_2892644
Anti-mouse IgG H&L antibody	Beyotime, China	A0216; RRID: AB_2860575
VeriBlot for IP Detection (HRP)	abcam	ab131366; RRID: AB_2892718
Anti-human CD34 microbeads	Miltenyi Biotec	130-046-702
Anti-human CD45 microbeads	Miltenyi Biotec	130-045-801
APC anti-human CD45 monoclonal antibody (clone HI30)	Biolegend	304037; RRID: AB_2562049
**Chemicals, peptides, and recombinant proteins**		
Cytarabine	Cytosar	N/A
Daunorubicin	New Era Pharmaceutical, Shangdong, China	N/A
NACET	MedChemExpress	HY-134495
PEITC	Macklin, China	P815538-5g
CCK-8 kit	Dojindo	CK04
Fixable Viability Dye eFluor 450	eBioscience	65-0863-18
CellROX Deep Red	Thermo Fisher Scientific	C10491
MitoSOX Red	Thermo Fisher Scientific	M36008
Calbryte 630 AM	AAT Bioquest	20720
Rhod-2	Thermo Fisher Scientific	R1245MP
BAPTA AM	MedChemExpress	HY-100545
20% Pluronic F-127	Thermo Fisher Scientific	P3000MP
Probenecid	MedChemExpress	HY-B0545
BzATP	MedChemExpress	HY-136254
AZ10606120	MedChemExpress	HY-108669
SN6	MedChemExpress	HY-107658
Ru360	Sigma-Aldrich	557440
CGP37157	MedChemExpress	HY-15754
mitoTEMPO	MedChemExpress	HY-112879
mitoPQ	MedChemExpress	Cat# HY-130278
PureProteome Protein A/G beads	Millipore	LSKMAGAG
MG-132	MedChemExpress	HY-13259
**Critical commercial assays**		
8-OHdG ELISA Kit	CUSABIO, Wuhan, China	CSB-E10140h
ABTS Kit	Beyotime, China	S0121
Genomic DNA Purification Kit	Promega	A1120
Dual-Luciferase Reporter Assay System	Promega	E1910
Annexin V-APC/7-AAD Apoptosis Detection Kit	KeyGen, Jiangsu, China	KGA1106-100
Reverse Transcription Kit	Thermo Fisher Scientific	K1622
Blastaq 2× qPCR MasterMix	Abm	G981

### Cell culture

All cell lines were maintained at 37 °C in a humidified incubator with 5% CO_2_. Human AML cell lines including U937 (RRID: CVCL_0007), HL-60 (RRID: CVCL_0002) and THP1 (RRID: CVCL_0006) were obtained from the Cell Bank of Type Culture Collection, Chinese Academy of Sciences (CAS; Shanghai, China), and cultured in RPMI-1640 medium (Gibco, Grand Island, NY, USA) supplemented with 10% fetal bovine serum (FBS; ABM). Human BMSCs (HS-5; RRID: CVCL_3720; ATCC, Manassas, VA, USA) and HEK293T cells (CAS; RRID: CVCL_0063) were cultured in high-glucose Dulbecco’s Modified Eagle’s Medium (DMEM; Gibco) supplemented with 10% FBS. All cell lines underwent short tandem repeat (STR) profiling for authentication and were routinely tested and confirmed to be free of mycoplasma contamination.

### Extraction of acute myeloid leukaemia blasts and bone marrow mesenchymal stem cells

This study was approved by the Committee for the Ethical Review of Research, Fujian Medical University Union Hospital (2023KJCX044). Human bone marrow aspirates were collected from patients with AML prior to treatment at Fujian Medical University Union Hospital, Fuzhou, China. Details on samples from patients with AML are shown in Table 2. AML blasts were purified using CD34 microbeads (Miltenyi Biotec, Bergish-Gladbach, Germany). The extraction of bone marrow mesenchymal stromal cells were carried out in accordance with previous reports^23^. Briefly, mononuclear cells (MNCs) were isolated using Ficoll Paque density centrifugation (TBD, Tianjin, China). MNCs were seeded with a density of 5 million cells/6-cm dish in RPMI1640 (Gibco) supplemented with 20% FBS (ABM). Non-adherent cells were removed and adherent bone marrow mesenchymal stem cells were passaged with a confluence of 80% and expanded up.

**Table 2:** Patient’s information.

No.	AML#1	AML#2	AML#3
**Gender/age (years)**	Male/51	Female/69	Male/53
**FAB type**	M5	M2a	M4Eo
**Bone marrow blasts**^a^ **(%)**	72.1	22.7	66.7
**WBC** **(10**^**9**^**/l)**	80.08	12.16	199.4
**Hb** **(g/l)**	56	62	61
**PLT** **(10**^**9**^**/l)**	5	22	16
**Cytogenetic profile**	46, XY[20]	46, XX[20]	46, XY, inv(16)(p13q22)[20]; CBFβ-MYH11
**Mutation status**	None	*IDH1*, *IDH2*, *TP53*, *CEBPA*	*NRAS*, *TET2*, *ASXL1*, *TP53*, *WT1*

^a^Proportion of bone marrow blasts at diagnosis. *FAB* French–American–British classification, *Hb* haemoglobin, *PLT* platelet, *WBC* white blood cell.

### In vitro co-culture of acute myeloid leukaemia cells with stromal cells

HS-5 cells or bone mesenchymal stem cells were seeded at a density of 1.0 × 10^5^ cells per well in six-well plates. Upon reaching ~70% confluence, the medium was replaced with 2 ml of complete RPMI-1640 medium containing 10% FBS and 5.0 × 10^5^ AML cells or 10.0 × 10^5^ AML blasts. After 24 h of co-culture, AML cells expressing human CD45 or CD34 were isolated using anti-human CD45/CD34 immunomagnetic beads (Miltenyi Biotec).

### CCK-8 cell viability assay

AML cells (2 × 10^4^/well) were seeded in 96-well plates and treated with cytarabine (Ara-C, Pharmacia, Italy), daunorubicin (DNR, Shandong New Era Pharmaceutical Co. Ltd, Linyi, China) or H_2_O_2_ (Sigma-Aldrich, St. Louis, Missouri, USA) for 24 h. Cell viability was assessed using the Cell Counting Kit-8 (Dojindo, Tokyo, Japan) as per the manufacturer’s protocol. After 4-h incubation at 37 °C, absorbance was measured at 450 nm and 630 nm using a SpectraMax i3X microplate reader (Molecular Devices, Silicon Valley, CA, USA). Growth inhibition rate was calculated as (mean OD_450__–630_ of negative control cells − mean OD_450__–630_ of drug-treated cells) / (mean OD_450__–630_ of negative control cells − mean OD_450__–630_ of blank control) × 100%. Each condition was tested in triplicate and repeated in three independent experiments.

### Apoptosis assay

AML cells from co-culture or monoculture were harvested, and 5.0 × 10^5^ cells were stained with 5 μl Annexin V-APC and 5 μl 7-AAD (KeyGen, Jiangsu, China) for 15 min at room temperature in the dark. Apoptosis was analysed by flow cytometry (Celesta, BD Biosciences, Franklin Lake, NJ, USA) with 30,000 events per sample.

### Live/dead cell staining

Cells (5.0 × 10^5^) were stained with 0.5 μl Fixable Viability Dye eFluor 450 (eBioscience, San Diego, CA, USA) in 500 μL for 30 min on ice in the dark. Live (FVD^–^) and dead (FVD^+^) populations were analysed by flow cytometry (30,000 events/sample).

### Total antioxidant activity assay

The total levels of various antioxidant macromolecules, antioxidant small molecules and enzymes within a system represent the overall antioxidant activity of that system. AML cells were lysed by ultrasonication in PBS, and total antioxidant capacity was quantified using the Total Antioxidant Capacity Assay Kit (Beyotime, Shanghai, China). Absorbance at 414 nm was measured using a microplate reader. Each assay included two technical replicates and was independently repeated three times.

### 8-hydroxy-2’-deoxyguanosine (8-OHdG) ELISA assay

Following 24-h Ara-C exposure, genomic DNA was extracted using the Wizard Genomic DNA Purification Kit (Promega, Madison, WI, USA), and DNA content was quantified by NanoDrop (Thermo Fisher Scientific, Waltham, MA, USA). 8-OHdG levels were determined from 2 μg DNA using an ELISA kit (Cusabio, Wuhan, China) as per the manufacturer’s instructions, and absorbance at 450 nm was measured. Data are expressed as relative 8-OHdG levels, with untreated cells or cells infected with the empty vector are normalized to 1. Values are shown as mean ± standard error of the mean from technical triplicates.

### Intracellular and mitochondrial reactive oxygen species detection

AML cells (5.0 × 10^5^) were stained with CellROX Deep Red (500 nM, Thermo Fisher Scientific) or MitoSOX Red (3 μM, Thermo Fisher Scientific) in Hank’s buffer for 45 or 30 min, respectively, at 37 °C. After two washes, fluorescence was analysed by flow cytometry. Viable cells (FVD^–^) were gated, and 30,000 events were collected per sample.

### Intracellular and mitochondrial Ca^2+^ measurement

For cytosolic Ca^2+^, cells were incubated with Calbryte 630 AM (5 μM, AAT Bioquest, Sunnyvale, CA, USA) in Hank’s buffer containing 0.04% Pluronic F-127 (Thermo Fisher Scientific) and 1 mM probenecid (MCE, Monmouth Junction, NJ, USA) for 45 min. Intracellular Ca^2+^ was detected by flow cytometry. For calcium flux dynamics of AML cells, AML cells stained with Calbryte 630 AM were stimulated by BzATP and AZ10606120, and the changes of intracellular Ca^2+^ in the cells were recorded using a fluorescent microplate reader. For mitochondrial Ca^2+^, cells were stained with 5 μM Rhod-2 AM (Thermo Fisher Scientific) under similar conditions, then incubated for 6 h in complete medium to clear cytoplasmic dye. Mitochondrial Ca^2+^ levels were measured by a microplate reader. Data are presented as relative fluorescence intensity, with untreated cells or cells infected with the empty vector normalized to 1.

### Western blot analysis

Whole-cell lysates were prepared in RIPA buffer containing PMSF, phosphatase and protease inhibitors, then denatured in 5× SDS buffer. Proteins were separated by SDS-PAGE, transferred to 0.45-μm PVDF membranes (Millipore, Boston, MA, USA), blocked with 8%–10% milk, and incubated with primary antibodies overnight (details on antibody information are shown in Table 1). HRP-conjugated secondary antibodies were applied, and signals detected using a Bio-Rad chemiluminescence system (Hercules, CA, USA). Band intensity was quantified using ImageJ.

### Generation of stable cell lines

The human P2RX7 overexpression plasmid (Ubi-MCS-SV40-puromycin) was obtained from GeneChem (Shanghai, China). sgRNAs targeting P2RX7, Nrf2, Keap1 and PGAM5 (all exon 2) were synthesized by Sangon Biotech (Shanghai, China) and cloned into the LentiCRISPRv2 vector (Addgene, Cambridge, MA, USA). sgRNA sequences were as follows:

(1) *P2RX7*: sg1: 5’-TGATGACAGGCTCTTTCCGC-3’; sg2: 5’-GTTGTGTCCCGAGGTAAGGA-3’; (2) *Nrf2*: sg1: 5’-GCGACGGAAAGAGTATGAGC -3’; (3) *Keap1*: sg1: 5’-GGTCAAGTACCAGGATGCAC-3’, sg2: 5’-TGTGTCCTCCACGTCATGAA-3’); (4) *PGAM5*: sg1: 5’-GAAGAGGAACGTGGAATCTG-3’.

HEK293T cells (CAS) were co-transfected with the target plasmid, psPAX2, and pMD2.G (ratio 4:3:1) using Lipofectamine 3000 (Thermo Fisher Scientific). Viral supernatants were harvested at 24 and 52 h, filtered (0.45 μm) and concentrated using 25% PEG 8000 in 0.75 M NaCl (1∶4 ratio) overnight at 4 °C, then centrifuged at 4000 × *g* for 20 min. Pellets were resuspended in growth media and stored at −80 °C. AML cells were transduced with the lentiviral particles and selected with puromycin (2.5 μg/ml; Solarbio, Shanghai, China) for 10 days. Knockout efficiency was confirmed by Western blot; overexpression was validated by real-time quantitative PCR (RT-qPCR) and Western blot.

### Real-time quantitative PCR

Total RNA was extracted with TRIzol (Thermo Fisher Scientific), and cDNA was synthesized using a Reverse Transcription Kit (Thermo Fisher Scientific). RT-qPCR was performed using Blastaq 2× qPCR MasterMix (ABM, Vancouver, Canada) on a 7500 system (Applied Biosystems, New York, USA). ACTB was used for normalization, and the 2^–ΔΔCt^ method was applied. Primer sequences were as follows:

(1) P2RX7:

forward: 5’- GGGCGGAATAATGGGCATTGAG -3’;

reverse: 5’- AAGGCGACGGAAACTGTATTTGG -3’.

(2) Nrf2:

forward: 5’- CACATCCAGTCAGAAACCAGTGG -3’;

reverse: 5’- GGAATGTCTGCGCCAAAAGCTG -3’.

(3) ACTB:

forward: 5’- CCAACCGCGAGAAGATGA -3’;

reverse: 5’- TCCATCACGATGCCAGTG -3’.

### Luciferase reporter assay

Three tandem copies of the antioxidant response element (ARE) sequence (5’-GTGACAAAGCAATCCCGTGACAAAGCAATCCCGTGACAAAGCAATAAAGCTTAGACACTAGAGGGTATATAATGGAAGCTCGACTTCCAG-3’) were cloned into a firefly luciferase reporter plasmid (F-luc, GeneChem). AML cells were co-transfected with equal amounts of the ARE-F-luc construct and a renilla luciferase plasmid (R-luc, GeneChem) to control for transfection efficiency. After 48 h, cells were co-cultured with HS-5 stromal cells for 24 h. Luciferase activity was measured using the Dual-Luciferase Reporter Assay System (Promega), and relative transcriptional activity was calculated as the ratio of firefly to renilla luciferase (F-luc/R-luc), normalized to a value of 1.

### Immunoprecipitation

Cells were lysed in NP-40 lysis buffer supplemented with PMSF and protease inhibitor cocktail for 30 min at 4 °C. Lysates were pre-cleared with 25 μl of protein A/G magnetic beads (Millipore) at room temperature, and then incubated with primary antibodies overnight at 4 °C with rotation. Subsequently, 50 μl of fresh beads were added and incubated for 30 min at room temperature. Bead-bound immunocomplexes were washed three times with 0.05% PBST and eluted in 5× SDS loading buffer. Eluates were analysed by Western blot.

### Ubiquitination assay

To assess Nrf2 ubiquitination, AML cells were lysed in NP-40 buffer containing PMSF and protease inhibitors. Endogenous Nrf2 was immunoprecipitated as described above, and ubiquitinated Nrf2 was detected by Western blot using an anti-ubiquitin antibody (Abcam, Cambridge, UK).

### Mice

All animal procedures were approved by the Animal Ethics Committee of Fujian Medical University (Protocol No. IACUC FJMU 2024-0279) and performed in accordance with institutional guidelines. 4-week-old male NOD/ShiLtJGpt-*Prkdc*^*em26Cd52*^*Il2rg* ^*em26Cd22*^/Gpt (NCG) mice and 8-week-old male C57BL/6 J mice were purchased from Guangdong Yakult Biotech Co., Ltd (China) and housed in a specific pathogen-free facility with ventilated cages. Mice were maintained under standard 12-h light/dark cycles with controlled temperature and humidity, and provided with autoclaved food and water ad libitum. Mice were randomly assigned to experimental groups. Each group comprised mice housed 5 per cage, with animal numbers indicated in the corresponding figure legends. Mice were monitored daily for signs of distress, and humane endpoints included ≥20% weight loss, hindlimb paralysis, severe hunching or limited mobility. All in vivo studies were conducted in the Animal Resources Center of Fujian Medical University.

### Acute myeloid leukaemia xenotransplantation in NOD/ShiLtJGpt-*Prkdc*^*em26Cd52*^*Il2rg*^*em26Cd22*^ /Gpt mice

AML xenografts were established by injecting 4.0 × 10^5^ AML cells suspended in 200 μl PBS into NCG mice via the tail vein. Two weeks post-injection, mice were randomly assigned to four treatment groups: vehicle control, Ara-C (20 mg/kg/day), AZ10606120 (10 mg/kg/day), or a combination of both agents. Treatments were administered intravenously once daily for 5 consecutive days. Mice were euthanized upon signs of terminal illness (e.g. hindlimb paralysis, coma). AML burden was quantified by flow cytometry using APC-conjugated anti-human CD45 antibody (Biolegend, San Diego, CA, USA) following mechanical dissociation and erythrocyte lysis of liver, spleen and bone marrow tissues. To assess antioxidant capacity in residual AML cells from bone marrow, human CD45^+^ cells were isolated using magnetic beads (Miltenyi Biotec), and total antioxidant activity and 8-OHdG levels were measured ex vivo.

### MLL-AF9 driven murine acute myeloid leukaemia model

MLL-AF9 AML cells were generated as previously described^24,25^. Briefly, the retroviral constructs MSCV-MLL-AF9-IRES-YFP (30 μg) along with the packaging plasmid pCL-ECO (15 μg) were co-transfected into HEK293T cells using Lipofectamine 3000 to produce the MLL-AF9 retrovirus. Lineage-negative foetal liver cells isolated from C57BL/6 J embryos were transduced with MLL-AF9 retrovirus and then intravenously injected into sublethal irradiation (6.5 Gy) C57BL/6 J mice (8–10 weeks old). YFP^+^ bone marrow cells were sorted from primary recipient mice by flow cytometry and transplanted into non-irradiated secondary recipients at a dose of 2.0 × 10^5^ cells per mouse. MLL-AF9 leukaemic mice of secondary recipients were administered vehicle, Ara-C (100 mg/kg/day), AZ10606120 (10 mg/kg/day), or a combination of both agents for 5 days via intraperitoneal injection. AML burden was analysed by flow cytometry and then YFP^+^ bone marrow cells were sorted to assess the antioxidant capacity.

### Statistical analysis

All statistical analyses were performed using SPSS version 25. Data distribution was tested for normality using the Shapiro–Wilk test. For normally distributed data, data are presented as mean ± standard error of the mean or standard deviation and compared using Student’s *t* test (for two groups) or one-way analysis of variance followed by Dunnett’s post hoc test (for multiple groups). For non-normally distributed data, data are presented as median with interquartile range and compared using the Mann–Whitney *U* test or Kruskal–Wallis test. Survival analysis was performed using Kaplan–Meier survival curves for the mice, and statistical significance was determined using the log-rank (Mantel–Cox) test. A *P* value < 0.05 was considered statistically significant.

## Results

### Bone marrow stromal cells promote chemoresistance by enhancing antioxidant capacity in acute myeloid leukaemia cells

**Fig. 1:**
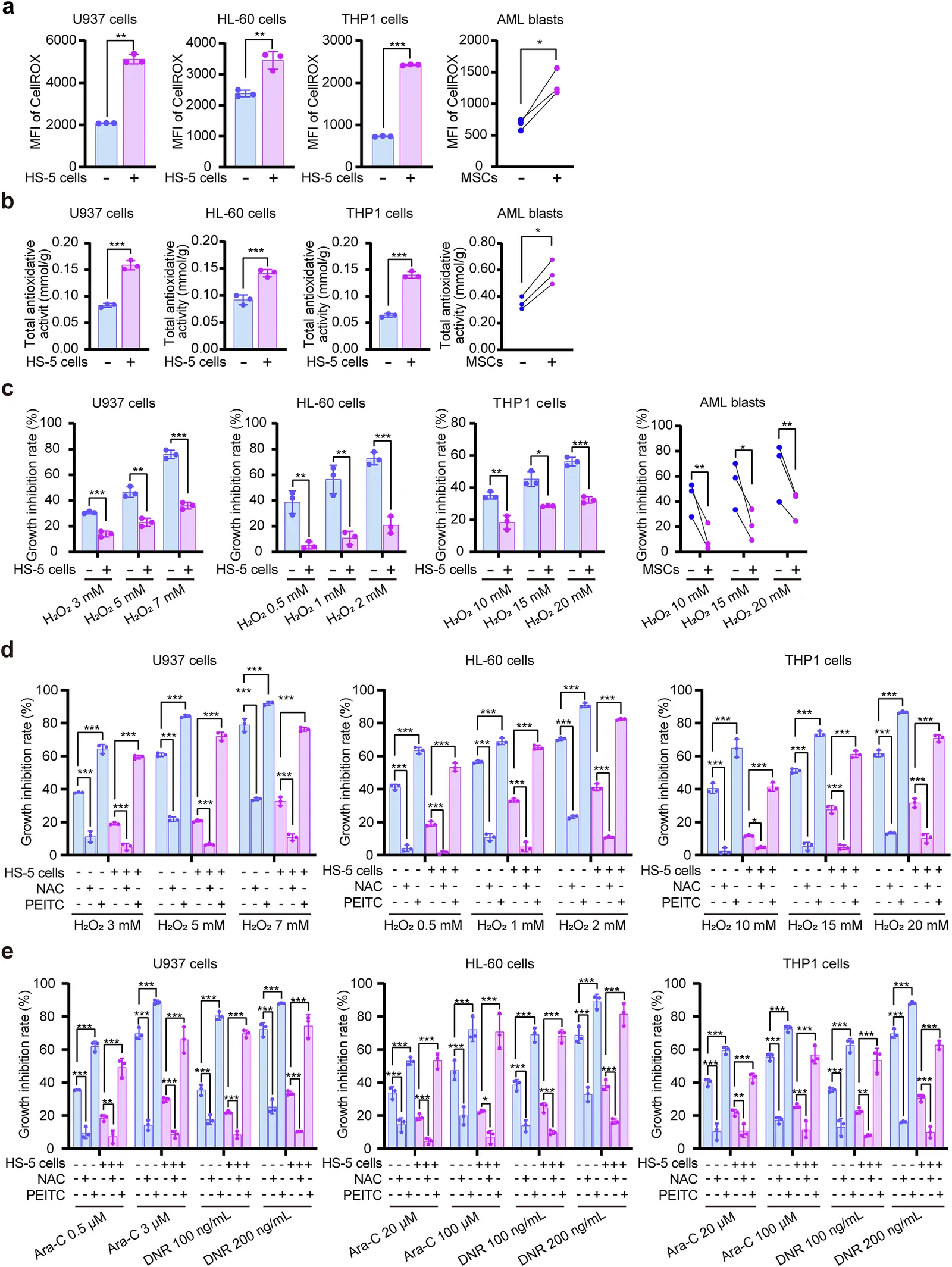
Bone marrow stromal cells promote chemoresistance by enhancing antioxidant capacity in acute myeloid leukaemia cells. **a** Quantification of intracellular reactive oxygen species in acute myeloid leukaemia (AML) cells and AML blasts co-cultured with or without bone marrow stromal cells, measured by CellROX fluorescence (*n* = 3 independent experiments). MFI, median fluorescence intensity, MSCs, mesenchymal stem cells. **b** Total antioxidant activity measured by ABTS assay in monocultured versus co-cultured AML cells (*n* = 3 independent experiments). **c** CCK-8 assay showing AML cell viability under exogenous H_2_O_2_ stress with or without co-culture (*n* = 3 independent experiments). **d** H_2_O_2_ tolerance of AML cells pretreated with N-acetyl-L-cysteine (NAC) (1 mM, 1 h) or 2-phenylethyl isothiocyanate (PEITC) (5 μM, 5 h), measured by CCK-8 (*n* = 3 independent experiments). **e** Chemosensitivity of AML cells pretreated as in **d** followed by 24-h cytarabine (Ara-C) or daunorubicin (DNR) exposure, assessed by CCK-8 (*n* = 3 independent experiments). Data are mean ± standard deviation. *P < 0.05, **P < 0.01, ***P < 0.001. See also Supplementary Fig. 1.

Our previous work demonstrated that AML cells co-cultured with HS-5 BMSCs exhibit increased resistance to Ara-C and DNR, two standard chemotherapeutic agents used in AML treatment, compared with AML cells cultured alone^4,11^. These findings suggest that stromal cells contribute to chemoresistance through mechanisms that remain to be fully defined. Notably, co-cultured AML cells displayed elevated mitochondrial oxidative phosphorylation (OXPHOS) activity, raising the possibility that increased mitochondrial metabolism may impact intracellular ROS levels. Indeed, staining with CellROX deep red dye revealed significantly higher intracellular ROS levels in co-cultured AML cells (Fig. 1a), indicative of a shift towards a more oxidative phenotype. Despite this increase in ROS, cell viability remained unchanged (Supplementary Fig. 1a–d), implying the presence of compensatory antioxidant defences induced by stromal support. Further in vitro analyses confirmed that co-cultured AML cells exhibited significantly greater total antioxidant activity (Fig. 1b) and increased resistance to hydrogen peroxide (H_2_O_2_)-induced oxidative stress (Fig. 1c) compared with monocultured cells, the phenomenon of which was also observed in primary AML cells. Treatment of AML cells with N-acetyl-L-cysteine (NAC), a glutathione (GSH) precursor, mimicked the stromal effect by enhancing antioxidant activity and chemoresistance (Fig. 1d, e and Supplementary Fig. 1e). In contrast, treatment with 2-phenylethyl isothiocyanate (PEITC), which depletes GSH, abrogated antioxidant function and sensitized AML cells to both H_2_O_2_ and chemotherapeutic agents (Fig. 1d, e and Supplementary Fig. 1e). Collectively, these data demonstrate that BMSCs enhance the antioxidant capacity of AML cells, thereby conferring resistance to oxidative stress and chemotherapy.

### P2RX7 upregulation is critical for enhancing antioxidant capacity in acute myeloid leukaemia cells

**Fig. 2:**
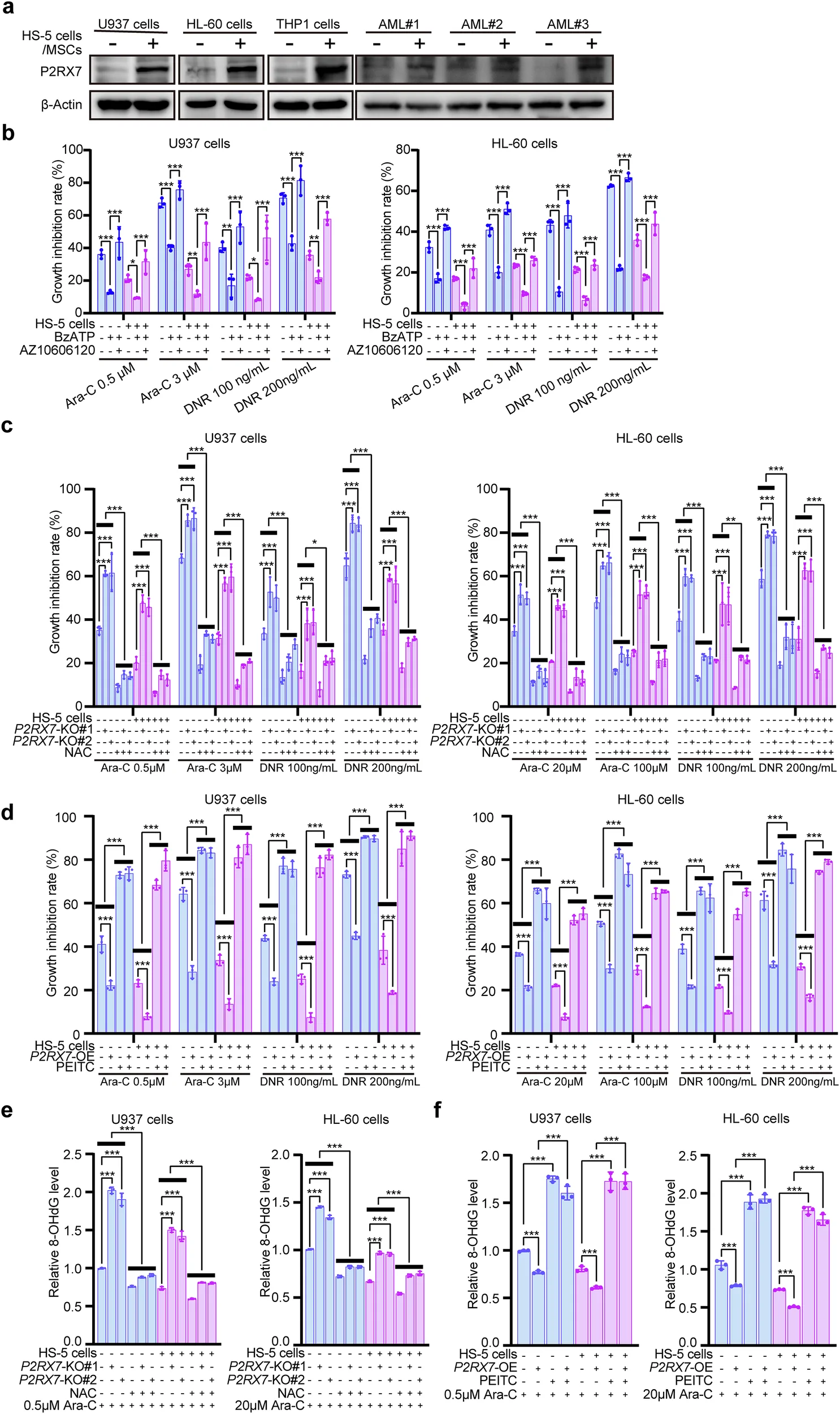
P2RX7 upregulation is critical for enhancing antioxidant capacity in acute myeloid leukaemia cells. **a** Western blot of P2RX7 protein in acute myeloid leukaemia (AML) cells and AML blasts with or without bone marrow stromal cells (MSCs) co-culture (*n* = 3 independent experiments). **b** CCK-8 assays showing chemosensitivity after 1-h treatment with BzATP (150 μM) or AZ10606120 (20 μM) (*n* = 3 independent experiments). **c**, **d** CCK-8 assays showing chemosensitivity of P2RX7-knockout AML cells treated with N-acetyl-L-cysteine (NAC) (1 mM, 1 h) (**c**) or of P2RX7-overexpressing cells treated with 2-phenylethyl isothiocyanate (PEITC) (5 μM, 5 h) (**d**) (*n* = 3 independent experiments). **e**, **f** 8-OHdG levels in P2RX7-KO (**e**) or P2RX7-OE (**f**) AML cells after cytarabine (Ara-C) treatment (24 h), with or without antioxidant modulation (*n* = 3 replicates). AML#1, Patient 1; AML#2, Patient 2; AML#3, Patient 3. Data are mean ± standard deviation (**b–d**) or mean ± standard error of the mean (**e**, **f**). *P < 0.05, **P < 0.01, ***P < 0.001. See also Supplementary Fig. 2.

Given the strong association between stromal support, enhanced antioxidant capacity and chemoresistance in AML, we next sought to identify upstream signalling mediators that govern this adaptive response. Our previous work showed that mitochondrial ATP production was significantly elevated in AML cells co-cultured with BMSCs, suggesting increased mitochondrial activity and potential engagement of ATP-dependent signalling pathways^11^. Among the candidate receptors, P2RX7 — a ligand-gated ion channel activated by ATP — stood out owing to its known involvement in regulating GSH metabolism^15^ and promoting survival in various tumour contexts^12,26,27^. We therefore investigated whether P2RX7 plays a role in BMSC-induced redox adaptation and chemoresistance in AML. Indeed, we observed a marked upregulation of P2RX7 protein expression in AML cells co-cultured with HS-5 cells compared with monocultured controls (Fig. 2a). To rule out the possibility that this effect is specific to HS-5 cells, we extracted bone marrow mesenchymal stem cells from AML patients and co-cultured them with primary AML cells. The primary AML cells also showed an upregulation of P2RX7 expression after coculture (Fig. 2a), suggesting a role for P2RX7 in mediating stromal-induced cellular antioxidant adaptation of AML cells. To assess the functional relevance of P2RX7 in chemoresistance, AML cells were treated with the P2RX7 agonist BzATP, which significantly reduced their sensitivity to Ara-C and DNR (Fig. 2b). In contrast, pharmacological inhibition of P2RX7 using the selective antagonist AZ10606120 suppressed the effect of BzATP, sensitizing AML cells to chemotherapy and partially reversing the protective effect conferred by stromal co-culture (Fig. 2b). To further validate the role of P2RX7, we generated AML cell lines with CRISPR/Cas9-mediated knockout or lentiviral overexpression of P2RX7 (Supplementary Fig. 2a–c). Genetic ablation of P2RX7 impaired both antioxidant capacity and chemoresistance, effects that were rescued by supplementation with GSH precursor NAC (Fig. 2c and Supplementary Fig. 2d, e). Conversely, overexpression of P2RX7 enhanced antioxidant capacity and chemoresistance, which could be abrogated by treatment with GSH-depleting agent PEITC (Fig. 2d and Supplementary Fig. 2f, g).

To directly link P2RX7-mediated antioxidant defence to protection against oxidative damage, we measured 8-hydroxy-2’-deoxyguanosine (8-OHdG), a marker of oxidative DNA damage. Ara-C treatment significantly increased 8-OHdG levels in AML cells (Supplementary Fig. 2h), and this effect was further augmented in P2RX7-deficient cells (Fig. 2e). Notably, co-culture with HS-5 cells failed to reduce 8-OHdG accumulation in P2RX7-null AML cells (Fig. 2e), indicating that stromal support is insufficient to compensate for P2RX7 loss. In contrast, P2RX7-overexpressing AML cells displayed reduced 8-OHdG levels following Ara-C exposure (Fig. 2f), reinforcing the role of P2RX7 in mitigating chemotherapy-induced oxidative stress. Together, these findings establish that BMSCs enhance antioxidant capacity and chemoresistance in AML cells, at least in part, through upregulation of P2RX7.

### P2RX7 imposes mitochondrial stress to licence a robust antioxidant programme in acute myeloid leukaemia cells

**Fig. 3:**
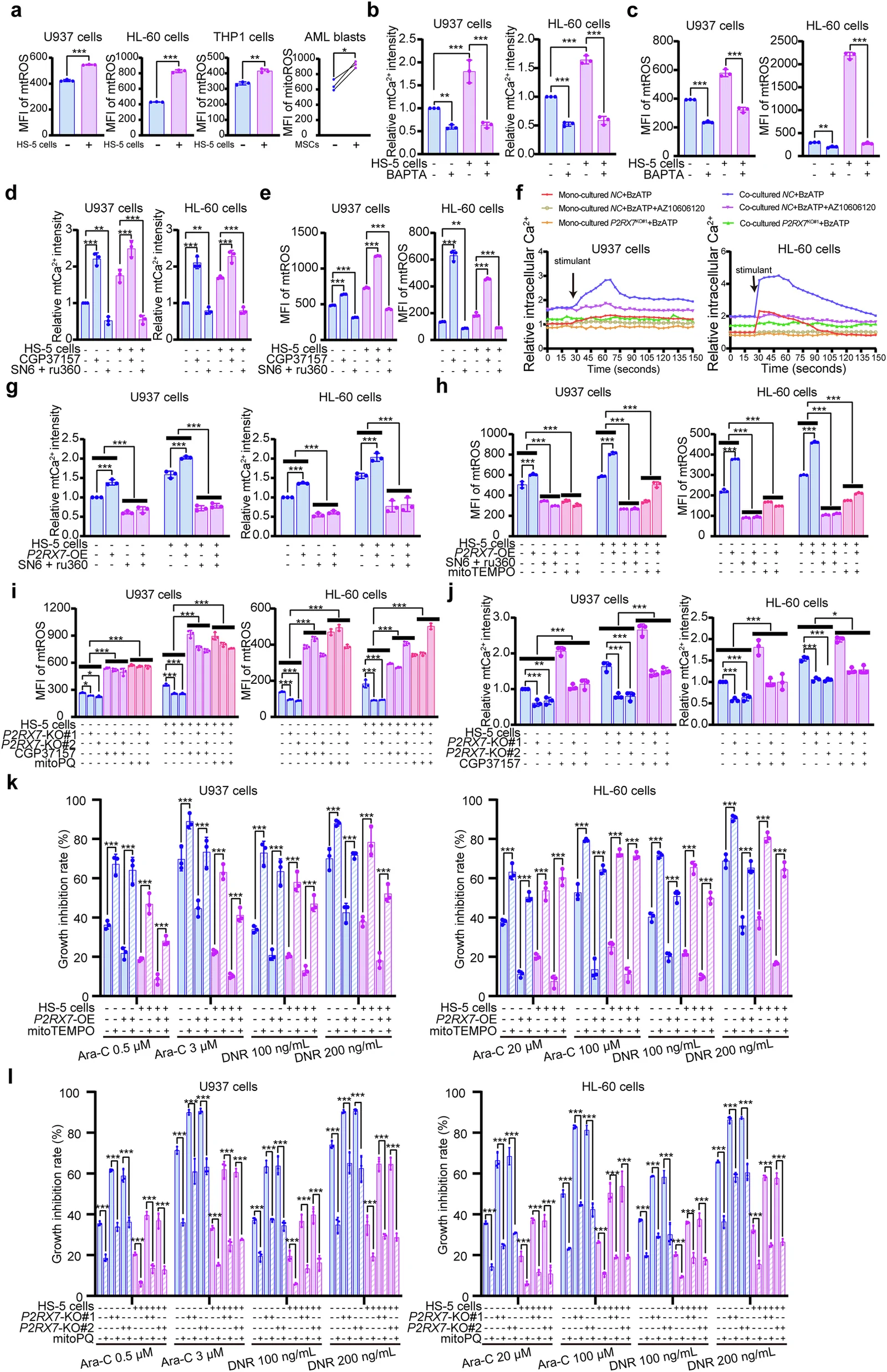
P2RX7 imposes mitochondrial stress to license a robust antioxidant programme in acute myeloid leukaemia cells. **a** Mitochondrial reactive oxygen species (mtROS) levels in acute myeloid leukaemia (AML) cells co-cultured with or without bone marrow stromal cells, measured by MitoSOX (*n* = 3 independent experiments). **b**, **c** Quantification of mitochondrial Ca^2+^ (mtCa^2+^) (**b**) and mtROS (**c**) in AML cells treated with BAPTA (20 μM, 6 h) (*n* = 3 independent experiments). Relative mtCa^2+^ intensity in the untreated cells was normalized to 1 (**b**). **d**, **e** mtCa^2+^ (**d**) and mtROS (**e**) levels after treatment with CGP37157 (5 μM), SN6 (20 μM) + Ru360 (20 μM) for 1 h (*n* = 3 independent experiments). Relative mtCa^2+^ intensity in the untreated cells was normalized to 1 (**d**). **f** Intracellular Ca^2+^ dynamics after BzATP (150 μM) or AZ10606120 (20 μM) stimulation, measured by Calbryte 630 AM (*n* = 3 independent experiments). **g**, **h** mtCa^2+^ (**g**) and mtROS (**h**) levels in P2RX7-overexpressing AML cells with or without SN6/Ru360 or mitoTEMPO (100 μM, 1 h) (*n* = 3 independent experiments). Relative mtCa^2+^ intensity in the untreated cells was normalized to 1 (**g**). **i**, **j** mtROS (**i**) and mtCa^2+^ (**j**) levels in P2RX7-knockout AML cells treated with CGP37157 or mitoPQ (20 μM, 1 h) (*n* = 3 independent experiments). Relative mtCa^2+^ intensity in the untreated cells was normalized to 1 (**j**). **k**, **l** CCK-8 assays showing chemosensitivity in P2RX7-overexpressing cells treated with mitoTEMPO (**k**) and P2RX7-knockout cells treated with mitoPQ (**l**) (*n* = 3 independent experiments). Data are mean ± standard deviation. *P < 0.05, **P < 0.01, ***P < 0.001. See also Supplementary Fig. 3.

To elucidate the mechanism by which P2RX7 enhances antioxidant capacity in AML cells, we first examined mtROS levels in co-cultured versus monocultured AML cells. Co-culture with BMSCs led to a significant elevation in mtROS levels (Fig. 3a). Given that P2RX7 functions as a calcium-permeable ion channel, we hypothesized that P2RX7 activation promotes cytosolic Ca^2+^ influx, which is subsequently transferred into the mitochondria, triggering mitochondrial oxidative stress and adaptive antioxidant responses. To test this, we modulated intracellular Ca^2+^ levels using BAPTA-AM, a cell-permeable Ca^2+^ chelator. BAPTA treatment reduced cytosolic Ca^2+^ levels (Supplementary Fig. 3a, b), which in turn decreased both mitochondrial Ca^2+^ (mtCa^2+^) uptake (Fig. 3b) and mtROS levels (Fig. 3c), supporting the role of calcium signalling in regulating mitochondrial redox status. To further investigate this relationship, we treated AML cells with SN-6 and Ru360 (inhibitors of mitochondrial Ca^2+^ uptake), or CGP37157 (an agonist of mitochondrial calcium uniporter, MCU). As expected, SN-6 and Ru360 suppressed mtCa^2+^ and mtROS levels, whereas CGP37157 increased both (Fig. 3d, e), demonstrating that mitochondrial calcium loading is a key determinant of mtROS production in AML cells.

Next, we directly evaluated the involvement of P2RX7 in regulating mtCa^2+^ and mtROS. Using Calbryte 630 AM dye, we measured real-time changes in cytosolic Ca^2+^ levels following P2RX7 activation. BzATP, a specific P2RX7 agonist, induced rapid Ca^2+^ influx, whereas the P2RX7 antagonist AZ10606120 effectively blocked this response (Fig. 3f). Of note, BzATP failed to elicit any rise in intracellular Ca^2+^ of P2RX7-null cells (Fig. 3f), confirming that the Ca^2+^ signal observed in control AML cells is P2RX7 dependent. Furthermore, P2RX7 overexpression enhanced mtCa^2+^ accumulation and mtROS levels, effects that were abolished by SN-6 and Ru360 treatment (Fig. 3g, h). In contrast, P2RX7 knockout diminished BMSC-induced increases in mtCa^2+^ and mtROS, which could be restored by MCU activation with CGP37157 (Fig. 3i, j). These results confirm that P2RX7 upregulation by BMSCs drives mitochondrial calcium overload and oxidative stress in AML cells.

To determine whether P2RX7-induced mitochondrial stress is responsible for the observed enhancement in antioxidant capacity, we treated P2RX7-overexpressing AML cells with mitoTEMPO, a mitochondria-targeted ROS scavenger. MitoTEMPO treatment reduced mtROS levels (Fig. 3h) and sensitized AML cells to both H_2_O_2_-induced oxidative stress (Supplementary Fig. 3c) and chemotherapeutic agents (Fig. 3k). Conversely, administration of mitoPQ, a mitochondria-targeted pro-oxidant, restored mtROS levels (Fig. 3i), antioxidant activity and chemoresistance in P2RX7-deficient AML cells (Fig. 3l and Supplementary Fig. 3d). Collectively, these findings demonstrate that stromal cell-induced upregulation of P2RX7 promotes mitochondrial calcium influx and oxidative stress, which in turn activates adaptive antioxidant defences that contribute to chemoresistance in AML cells.

### P2RX7-driven mitochondrial oxidative stress promotes Nrf2 accumulation and activation

**Fig. 4:**
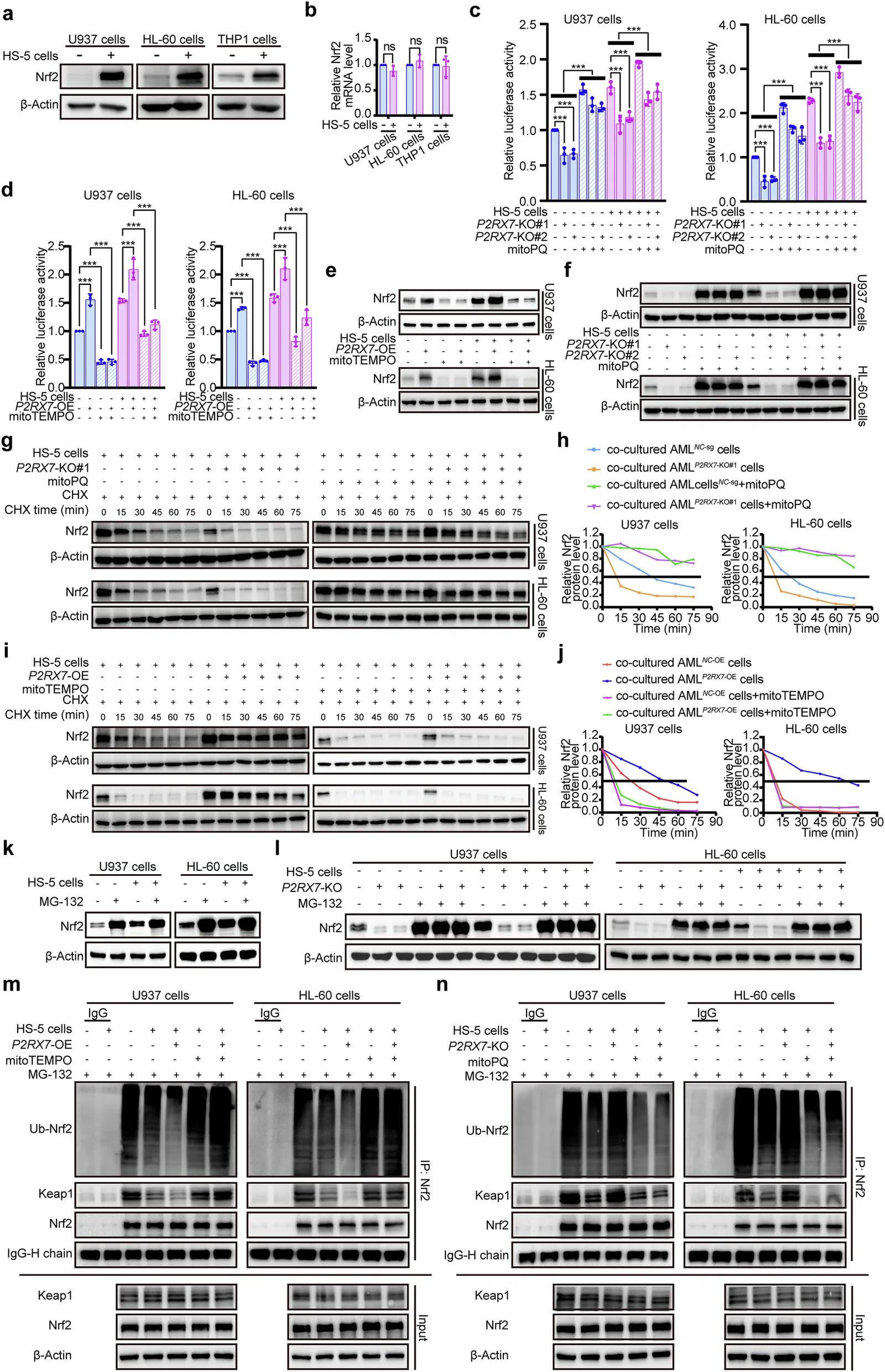
P2RX7-driven mitochondrial oxidative stress promotes Nrf2 accumulation and activation. **a**, **b** Western blot (**a**) and real-time quantitative PCR (RT-qPCR) (**b**) showing Nrf2 protein and messenger RNA (mRNA) levels in acute myeloid leukaemia (AML) cells with or without HS-5 co-culture (*n* = 3 independent experiments). **c**, **d** ARE-luciferase reporter activity in P2RX7-KO (**c**) or P2RX7-OE (**d**) AML cells treated with mitoPQ (20 μM) or mitoTEMPO (100 μM), respectively (*n* = 3 independent experiments). Relative luciferase activity in the untreated cells was normalized to 1. **e**, **f** Nrf2 protein levels assessed by Western blot in P2RX7-OE (**e**) and P2RX7-KO (**f**) AML cells following antioxidant modulation (*n* = 3 independent experiments). **g**, **h** Western blot (**g**) and ImageJ quantification (**h**) of Nrf2 degradation kinetics in P2RX7-KO AML cells treated with cycloheximide (CHX) (100 μg/ml) ± mitoPQ (*n* = 3 independent experiments). **i**, **j** Western blot (**i**) and ImageJ quantification (**j**) of Nrf2 in P2RX7-OE cells treated with CHX ± mitoTEMPO (*n* = 3 independent experiments). **k**, **l** Nrf2 protein levels in AML cells (**k**) and in P2RX7-KO cells (**l**) ± MG-132 (10 μM, 6 h) (*n* = 3 independent experiments). **m**, **n** Immunoprecipitation (IP) of Nrf2 followed by Western blot to assess Ub-Nrf2 levels and Nrf2-Keap1 binding in P2RX7-OE (**m**) or P2RX7-KO (**n**) AML cells, with mitoTEMPO or mitoPQ, respectively (*n* = 3 independent experiments). Data are mean ± standard deviation. *P < 0.05, **P < 0.01, ***P < 0.001; ns not significant. See also Supplementary Fig. 4.

Nrf2 is a master transcriptional regulator of antioxidant responses. In AML cells co-cultured with HS-5 stromal cells, Nrf2 protein levels were significantly elevated, whereas its messenger RNA (mRNA) expression remained unchanged (Fig. 4a, b), suggesting post-transcriptional regulation. To investigate the mechanism by which P2RX7 mediates BMSC-induced antioxidant capacity, we explored the relationship between P2RX7 signalling and Nrf2 activation. Notably, genetic ablation of Nrf2 abolished the ability of P2RX7 overexpression to enhance antioxidant capacity and chemoresistance in AML cells (Supplementary Fig. 4a–d), indicating that the protective effects conferred by P2RX7 are Nrf2 dependent. As Nrf2 activates antioxidant gene expression by binding to antioxidant response elements (AREs) in their promoters, we assessed its transcriptional activity using a luciferase reporter assay. P2RX7 knockout significantly impaired co-culture-induced ARE-driven luciferase activity (Fig. 4c), whereas P2RX7 overexpression enhanced basal Nrf2 transcriptional activity (Fig. 4d). This effect was suppressed by mitoTEMPO, a mitochondria-targeted antioxidant, indicating that mitochondrial oxidative stress is required for P2RX7-mediated Nrf2 activation (Fig. 4d).

We next examined how P2RX7 regulates Nrf2 protein expression. Overexpression of P2RX7 robustly increased Nrf2 protein levels, whereas mitoTEMPO abrogated this effect (Fig. 4e). Conversely, P2RX7 knockout reduced Nrf2 protein expression, which could be restored by the mitochondria-targeted oxidant mitoPQ (Fig. 4f). In all cases, Nrf2 mRNA levels remained unchanged (Supplementary Fig. 4e, f), suggesting that P2RX7 regulates Nrf2 primarily at the post-translational level. To test whether P2RX7 affects Nrf2 protein stability, we treated AML cells with cycloheximide (CHX), a protein synthesis inhibitor, and monitored the time-dependent degradation of Nrf2. In co-cultured P2RX7-knockout AML cells, Nrf2 degradation was accelerated, but could be rescued by mitoPQ (Fig. 4g, h). In contrast, Nrf2 degradation was slower in P2RX7-overexpressing cells, and mitoTEMPO treatment accelerated its degradation (Fig. 4i, j). These results indicate that P2RX7-driven mitochondrial stress enhances Nrf2 protein stability under co-culture conditions.

As Nrf2 is primarily regulated by ubiquitin-proteasome-mediated degradation^28,29^, we examined whether P2RX7 interferes with this process. Treatment with the proteasome inhibitor MG-132 increased Nrf2 protein levels in monocultured AML cells (Fig. 4k) and completely restored Nrf2 expression in P2RX7-deficient cells (Fig. 4l), implicating the proteasomal pathway. To directly assess Nrf2 ubiquitination, we performed immunoprecipitation in AML cells treated with MG-132. P2RX7 overexpression markedly reduced the ubiquitination level of Nrf2, whereas P2RX7 knockout increased it. Moreover, modulation of mtROS levels by mitoPQ or mitoTEMPO correspondingly decreased or increased Nrf2 ubiquitination (Fig. 4m, n). Collectively, these findings demonstrate that P2RX7-driven mitochondrial oxidative stress stabilizes Nrf2 by inhibiting its ubiquitin-proteasome-mediated degradation, thereby activating the Nrf2-ARE signalling axis and enhancing the antioxidant defence programme in AML cells.

### P2RX7–PGAM5 signalling couples mitochondrial stress to Nrf2-driven chemoresistance in AML cells

A central question arising from our findings is how P2RX7-driven mitochondrial oxidative stress transmits signals to activate Nrf2. It is well established that Nrf2 is tightly regulated by Keap1-dependent ubiquitin-proteasome degradation, which is controlled through direct binding between Keap1 and Nrf2 (refs.^17,30^). To assess whether P2RX7 affects this interaction, we immunoprecipitated Nrf2 from AML cells treated with the proteasome inhibitor MG-132. Overexpression of P2RX7 significantly reduced the binding between Nrf2 and Keap1, whereas treatment with mitoTEMPO restored this interaction (Fig. 4m). Conversely, P2RX7 knockout enhanced Keap1–Nrf2 complex formation, which was reversed by the mitochondria-targeted pro-oxidant mitoPQ (Fig. 4n). These results indicate that, under stromal co-culture conditions, P2RX7-induced mitochondrial stress disrupts Keap1–Nrf2 complex formation, thereby promoting Nrf2 stabilization.

**Fig. 5:**
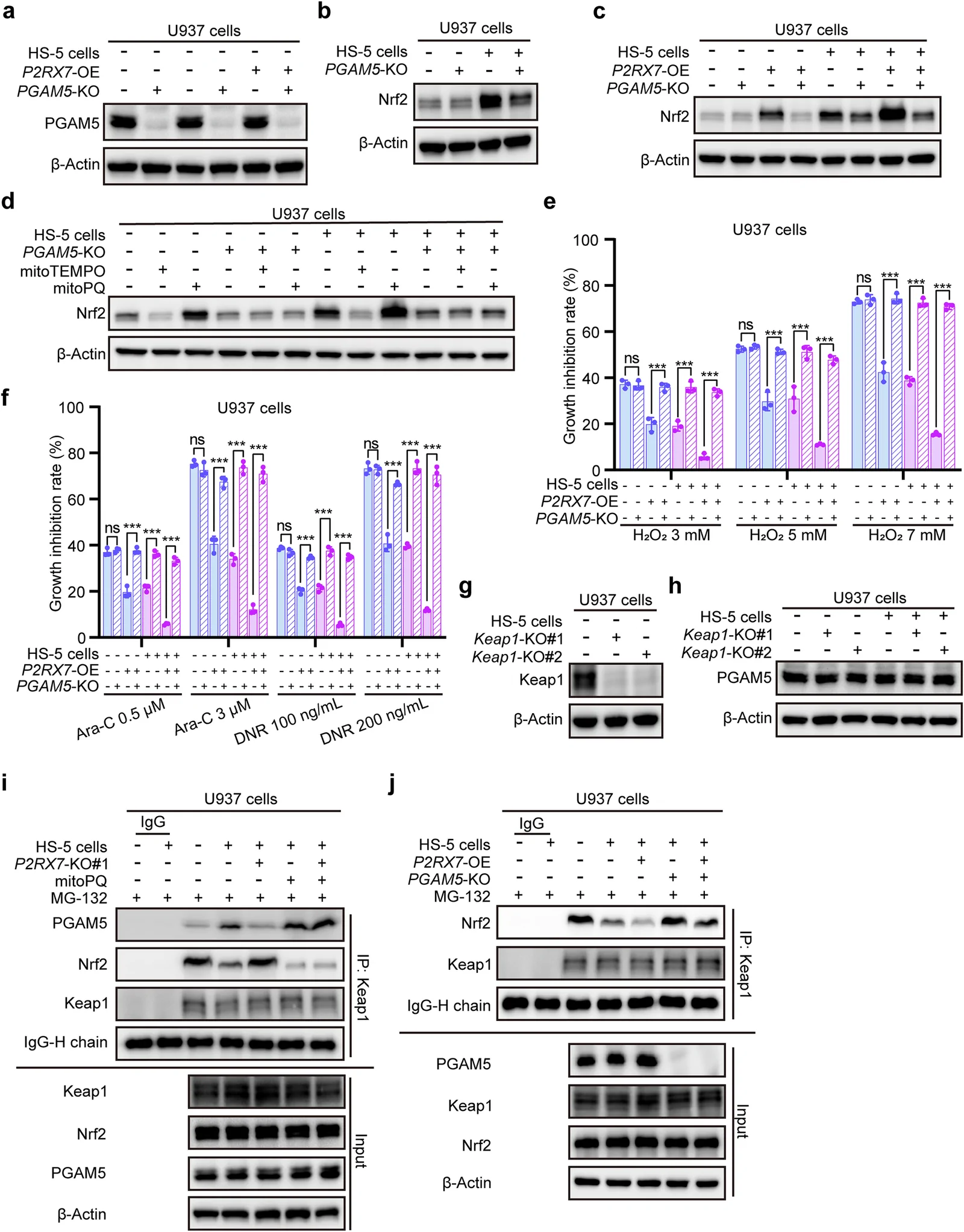
P2RX7–PGAM5 signalling couples mitochondrial stress to Nrf2-driven chemoresistance in acute myeloid leukaemia cells. **a** PGAM5 knockout efficiency in acute myeloid leukaemia (AML) cells ± P2RX7 overexpression (*n* = 3 independent experiments). **b–d** Nrf2 protein levels in PGAM5-KO cells under HS-5 co-culture (**b**), P2RX7 overexpression (**c**) or mitoTEMPO/mitoPQ treatment (**d**), and heatmap showing relative Nrf2 protein abundance from Western blot analysis (*n* = 3 independent experiments). **e**, **f** H_2_O_2_ tolerance (**e**) and chemosensitivity (**f**) in P2RX7-OE cells ± PGAM5 knockout (*n* = 3 independent experiments). **g**, **h** Keap1 knockout efficiency (**g**) and PGAM5 protein levels in Keap1-KO cells (**h**) (*n* = 3 independent experiments). **i**, **j** Co-IP of Keap1 followed by Western blot to assess its interaction with Nrf2 or PGAM5 in P2RX7-KO (**i**) or P2RX7-OE (**j**) cells, with/without PGAM5 knockout and MG-132 (*n* = 2 independent experiments). Data are mean ± standard deviation. *P < 0.05, **P < 0.01, ***P < 0.001; ns not significant.

We next examined the role of PGAM5, a mitochondrial membrane-anchored protein known to function as a stress signal transducer. To determine whether PGAM5 mediates antioxidant responses triggered by P2RX7, we knocked out PGAM5 in U937 AML cells. PGAM5 loss did not affect basal Nrf2 protein levels in monocultured cells, but it markedly attenuated the increase in Nrf2 protein observed in co-cultured cells (Fig. 5a, b). Moreover, P2RX7 overexpression failed to induce Nrf2 accumulation in PGAM5-deficient cells (Fig. 5c). To further evaluate whether mtROS regulates Nrf2 in a PGAM5-dependent manner, we modulated mtROS using mitoTEMPO or mitoPQ in PGAM5-knockout cells. Neither treatment altered Nrf2 levels (Fig. 5d), suggesting that PGAM5 is essential for transducing mtROS signals to Nrf2. Consistent with this, PGAM5 deletion also abolished the ability of P2RX7 to enhance antioxidant capacity and chemoresistance in AML cells (Fig. 5e, f), aligning with the changes in Nrf2 protein levels.

Previous studies suggest that Keap1 may mediate the ubiquitination and degradation of PGAM5 (ref.^21^). However, in Keap1-knockout AML cells, PGAM5 protein levels remained unchanged (Fig. 5g, h), indicating that Keap1 does not significantly regulate PGAM5 stability in this context. Based on these findings, we hypothesized that PGAM5 may facilitate Nrf2 activation by interacting with Keap1 and disrupting the Keap1–Nrf2 complex under conditions of mitochondrial oxidative stress. Indeed, co-immunoprecipitation revealed that stromal co-culture promoted the interaction between PGAM5 and Keap1, whereas P2RX7 knockout impaired this interaction (Fig. 5i). Importantly, mitoPQ restored PGAM5–Keap1 binding in the absence of P2RX7 (Fig. 5i). Furthermore, in PGAM5-deficient cells, P2RX7 overexpression was unable to disrupt the Keap1–Nrf2 complex (Fig. 5j), reinforcing the requirement of PGAM5 in this regulatory axis. Collectively, these findings demonstrate that PGAM5 acts as a critical mediator of mitochondria-to-nucleus crosstalk by relaying P2RX7-induced mitochondrial stress signals to the Keap1–Nrf2 axis. This pathway ultimately drives Nrf2 stabilization and activation, thereby orchestrating the antioxidant defence programme that contributes to AML chemoresistance under stromal influence.

### P2RX7 antagonism synergizes with cytarabine to attenuate leukaemic burden in vivo

**Fig. 6:**
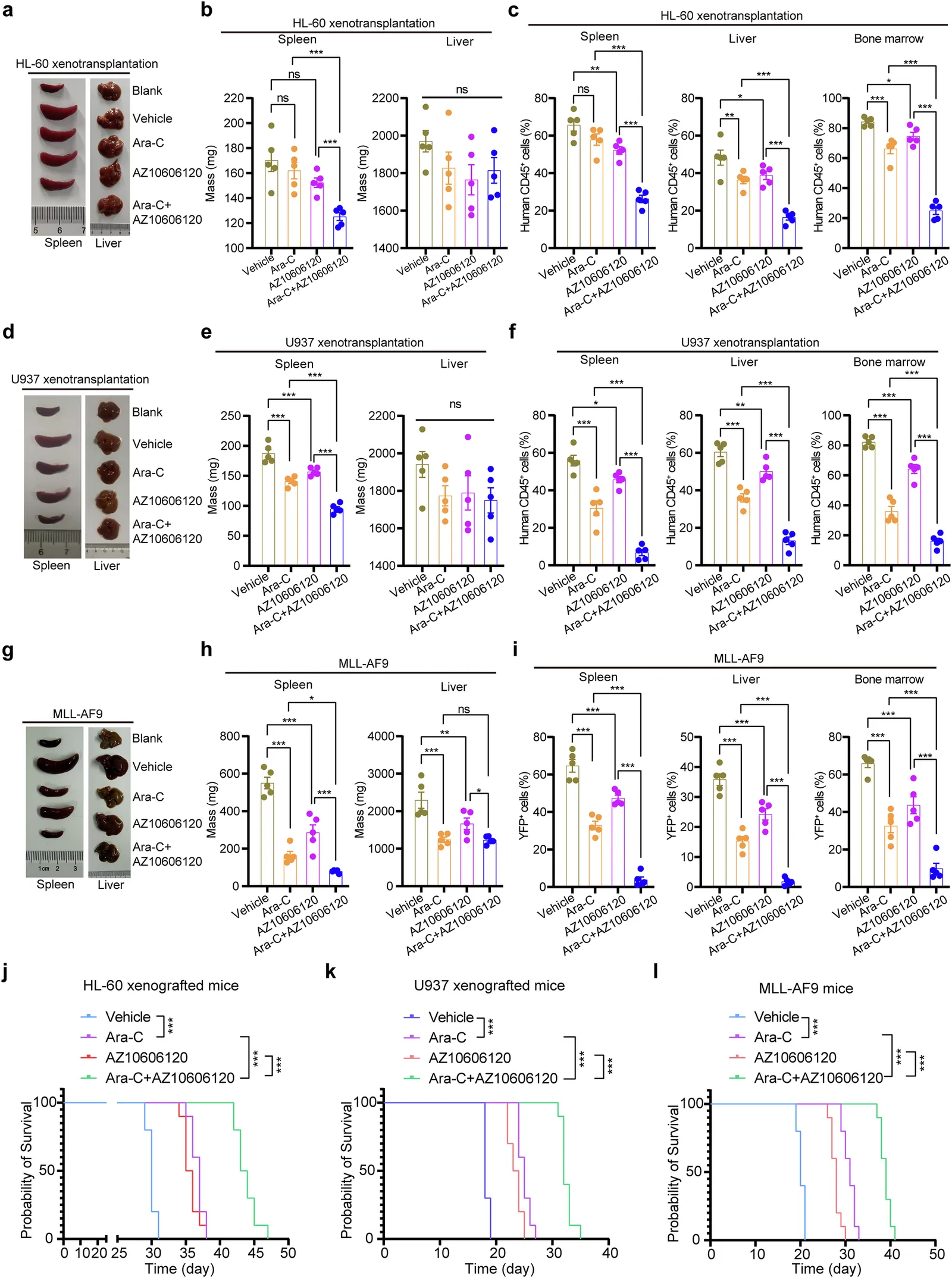
P2RX7 antagonism synergizes with cytarabine to attenuate leukaemic burden in vivo. HL-60, U937 or murine MLL-AF9 acute myeloid leukaemia (AML) cells were injected into mice via the tail vein. After 2 weeks, mice received vehicle, cytarabine (Ara-C), AZ10606120 or combination for 5 days. **a**, **d**, **g** Representative images of spleen and liver from HL-60 xenografted mice (**a**), U937 xenografted mice (d) and murine MLL-AF9 mice (**g**). **b**, **e**, **h** Organ weights (*n* = 5 biological replicates). **c**, **f**, **i** Frequencies of AML cells in spleen, liver and bone marrow (*n* = 5). **j**, **k**, **l** Survival curves of each group (*n* = 10 biological replicates). Data are mean ± standard error of the mean. *P < 0.05, **P < 0.01, ***P < 0.001l; ns not significant.

To evaluate whether the chemosensitizing effect of P2RX7 inhibition observed in vitro translates to therapeutic benefit in vivo, we established a systemic AML mouse model by injecting HL-60 or U937 cells into NCG mice via the tail vein. Mice were randomized into four treatment groups: vehicle control, P2RX7 antagonist AZ10606120 (10 mg/kg/day), Ara-C (20 mg/kg/day), and a combination of AZ10606120 and Ara-C. All treatments were administered via tail vein once daily for 5 consecutive days. In HL-60-engrafted mice, monotherapy with either Ara-C or AZ10606120 had minimal impact on hepatosplenomegaly. In contrast, combination therapy significantly reduced spleen size, although liver weights remained unchanged (Fig. 6a, b). Flow cytometry analysis (gating hierarchy shown in Supplementary Fig. 5a) revealed that the combination treatment markedly decreased AML cell infiltration in both extramedullary organs (spleen and liver) and bone marrow (Fig. 6c), indicating improved disease control. Similarly, in the U937 xenograft model, combined AZ10606120 and Ara-C treatment more effectively reduced splenomegaly (Fig. 6d, e) and substantially decreased leukaemic cell burden in the spleen, liver and bone marrow (Fig. 6f) compared with either monotherapy. In addition, we transplanted the MLL-AF9-driven murine AML cells into syngeneic C57BL/6 mice with a normal immune system and repeated the treatment protocol. The combined treatment significantly relieved hepatosplenomegaly (Fig. 6g, h). Flow cytometry also showed that the combined treatment significantly reduced the leukaemic burden (Fig. 6i). Even more, the combined treatment significantly prolonged the survival period of the mice (Fig. 6j–l). These in vivo results are consistent with our in vitro findings and demonstrate that pharmacological inhibition of P2RX7 enhances the therapeutic efficacy of Ara-C by overcoming chemoresistance and reducing leukaemic infiltration across multiple compartments.

### Impaired antioxidant capacity underlies P2RX7 antagonism-mediated potentiation of cytarabine in vivo

**Fig. 7:**
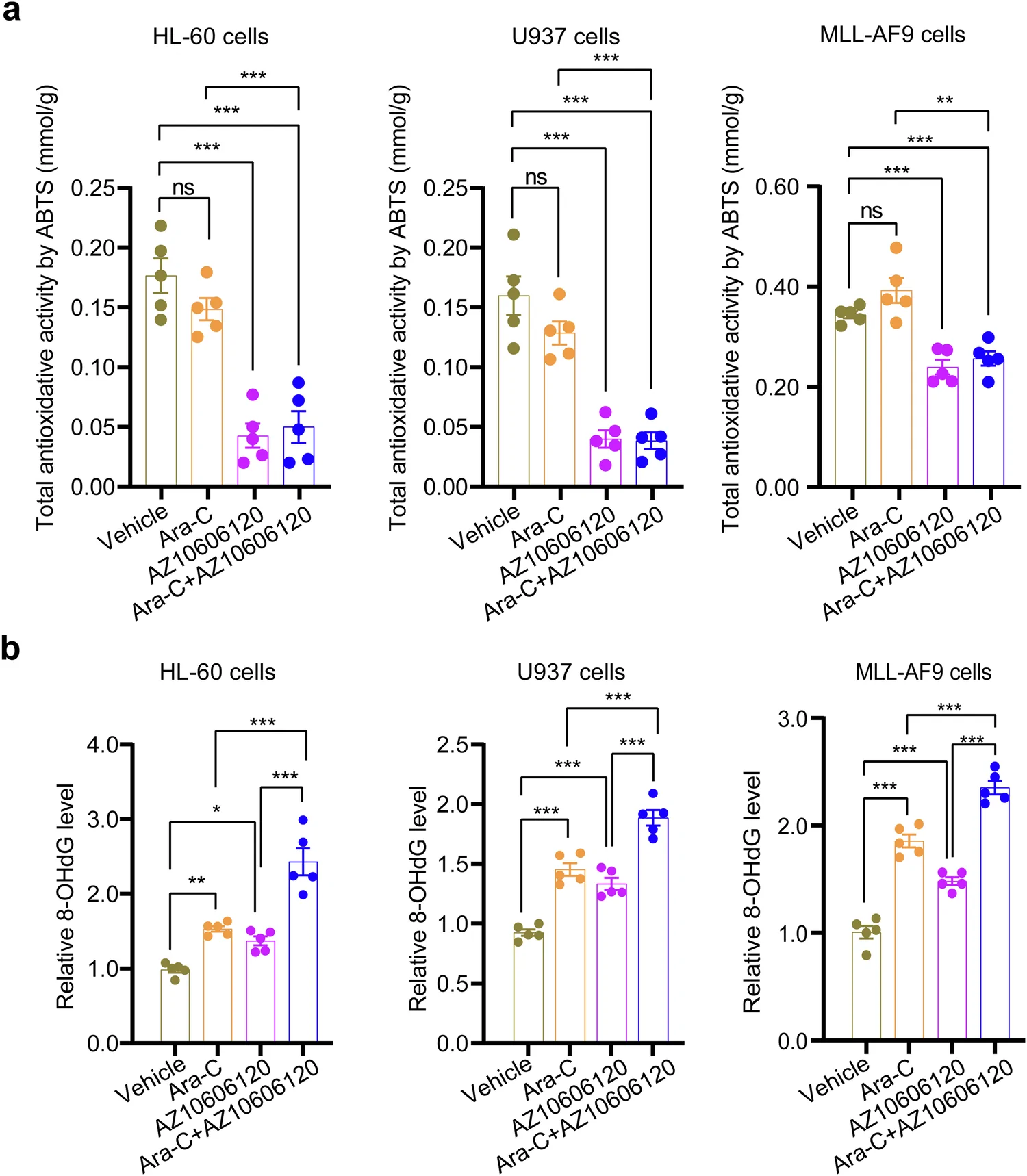
Impaired antioxidant capacity underlies P2RX7 antagonism-mediated potentiation of cytarabine in vivo. **a**, **b** Human CD45^+^ acute myeloid leukaemia cells or YFP^+^ cells were isolated from bone marrow of mice in Fig. 6. **a** Total antioxidant activity and **b**, 8-OHdG levels, were quantified (*n* = 5 biological replicates). Data are mean ± standard error of the mean. *P < 0.05, **P < 0.01, ***P < 0.001; Ara-C, cytarabine; ns not significant.

Building on the observation that the P2RX7 antagonist AZ10606120 potentiated the anti-leukaemic activity of Ara-C in vivo, we next investigated whether this effect was mediated through disruption of antioxidant defences in AML cells. Bone marrow tissues were harvested from xenografted mice following the various treatment regimens. Single-cell suspensions were prepared, and human AML cells or murine AML cells were isolated using anti-human CD45 immunomagnetic beads or FCM correspondingly. Total antioxidant capacity and oxidative DNA damage, measured by 8-OHdG levels, was assessed ex vivo. AZ10606120 monotherapy significantly reduced the total antioxidant capacity of AML cells and concomitantly increased 8-OHdG accumulation, indicative of elevated oxidative DNA damage. Notably, these effects were further exacerbated in the combination treatment group, where AZ10606120 was administered alongside Ara-C (Fig. 7a, b). These findings suggest that the enhanced therapeutic efficacy of Ara-C in vivo is at least partially attributable to the ability of AZ10606120 to compromise antioxidant defences, rendering AML cells more vulnerable to oxidative stress and chemotherapeutic damage.

## Discussion

AML is a malignancy with a strong dependence on the BMME, where BMSCs play a key role in promoting disease progression and drug resistance^31,32^. Emerging evidence suggests that one mechanism by which BMSCs contribute to chemoresistance is through enhancing the antioxidant capacity of AML cells^10^. However, the molecular basis for this protective effect has remained poorly defined.

In this study, we demonstrate that AML cells co-cultured with BMSCs exhibit elevated ROS levels without a corresponding decrease in viability, suggesting activation of adaptive antioxidant defences. Consistent with previous reports that highlight the role of BMSCs in regulating redox balance, such as promoting cysteine availability for GSH synthesis in chronic lymphocytic leukaemia^33^ and conferring GSH-dependent resistance in AML^10^, we show that antioxidant capacity is a key determinant of AML cell survival and chemoresistance. Our use of NAC and PEITC to modulate antioxidant capacity further supports this relationship, which raises important questions about the unintended consequences of antioxidant use in cancer therapy.

The mitochondria of AML cells exhibit abnormal metabolic reprogramming and elevated ROS level. Many studies have suggested that increased mtROS level can induce apoptosis^34,35^. As the adage goes, “What doesn’t kill me makes me stronger”, mild mitochondrial stress can trigger protective signalling pathways — a phenomenon known as mitohormesis^36^. This theory posits that moderate mitochondrial stress triggered by various factors can initiate a wide range of cellular protective states, the most classic of which is the mitochondrial unfolded protein response to maintain mitochondrial stability and the cellular protective antioxidant defences against mtROS damage. Our findings provide mechanistic insight into how BMSCs influence mitochondrial function and redox homeostasis in AML cells. In our model, BMSCs induced the expression of the purinergic receptor P2RX7 in AML cells, which led to mtCa^2+^ overload, increased mtROS production, and this mitochondrial stress enhanced antioxidant capacity and contributes to chemoresistance. These observations align with prior studies showing that mtCa^2+^ loading promotes OXPHOS and metabolic adaptation in AML^37^.

The role of P2RX7 in cancer has been reported to be context dependent. In some cancers, it promotes cell death^38^, in others, it supports tumour progression^39^. Our study adds to this complex picture by highlighting its function in mitochondrial signalling and redox regulation in AML. Specifically, P2RX7 activation triggers mitochondrial oxidative stress, which enhances the antioxidant response, thereby promoting chemoresistance. Importantly, pharmacological blockade of P2RX7 with AZ10606120 sensitized AML cells to Ara-C both in vitro and in vivo, an effect associated with impaired antioxidant defences and increased oxidative DNA damage. However, the clinical translation of P2RX7 inhibitors requires careful consideration of potential off-target effects, given its pleiotropic roles in immunity and normal tissue homeostasis. It is worth noting that we constructed the MLL-AF9 mouse AML model in C57BL/6 mice with normal immune systems and observed that the combination of P2RX7 antagonist AZ1006120 and Ara-C had significant effects in killing tumour cells and increasing the survival period of mice. The emergence of highly selective antagonists and advanced delivery strategies (e.g. liposomal systems and bone marrow-targeting modalities) could mitigate systemic exposure. Thus, by elucidating why AML cells exploit P2RX7 for survival, our work not only strengthens the rationale for its targeted inhibition but also provides a framework for its clinically viable application. Furthermore, it has reported that the ATP level in the bone marrow niche of AML mice is increased, which can activate P2RX7 to promote the homing and self-renewal capacities of leukaemia-initiating cells^14^. However, whether ATP is the sole activating ligand for P2RX7 in the leukaemic microenvironment remains unknown. Importantly, BMSCs are known to interact with AML cells through multiple bidirectional mechanisms — including direct cell–cell contact (e.g. via adhesion molecules and gap junctions), paracrine signalling (e.g. CXCL12, TGFβ, IL-6), and transfer of extracellular vesicles carrying proteins, lipids and nucleic acids^40,41^. These interactions can reprogramme AML cell metabolism, survival signalling and chemoresistance. It is therefore plausible that stromal cues may also regulate P2RX7 expression, membrane trafficking or activation in AML cells, by modulating extracellular nucleotide availability, by delivering P2RX7-regulatory molecules through extracellular vesicles or by engaging contact-dependent signalling pathways that converge on purinergic signalling. Conversely, P2RX7 activation in AML cells may further influence their crosstalk with BMSCs, creating a feed-forward loop that reinforces the protective leukaemic niche. Future studies should systematically dissect the upstream mechanisms controlling P2RX7 expression and activation in AML cells versus normal HSPCs within the remodelled BMSC niche, including the contribution of distinct stromal components and the potential cooperation between ATP and other niche-derived signals. Defining these context-dependent interactions may reveal additional therapeutic vulnerabilities for selectively targeting P2RX7-dependent chemoresistance in leukaemic cells while preserving normal haematopoiesis.

We further explored how mitochondrial stress is sensed and transduced to activate antioxidant pathways. Our results show that P2RX7-induced mtROS enhances the stability and transcriptional activity of Nrf2, a master regulator of the antioxidant response. Nrf2 protein, but not mRNA, was upregulated in co-cultured AML cells, indicating post-transcriptional regulation. Functional assays revealed that Nrf2 knockout abolished P2RX7-induced antioxidant activity and chemoresistance, establishing Nrf2 as an essential downstream effector. Beyond regulating total antioxidant capacity, known targets of Nrf2 (such as aldosterone reductase family members, Heme oxygenase-1 etc.) mediate pleiotropic protective effects in various cancers, including detoxification, anti-apoptosis and metabolic adaptation^42,43^. Consequently, the pathway we discovered probably coordinates a comprehensive survival programme, of which enhanced antioxidant capacity is just one component. Nrf2 activation may orchestrate a multifaceted adaptive response in AML cells.

Disruption of the Keap1–Nrf2 complex stabilizes Nrf2 protein and initiates antioxidant-defence programmes. Extensive studies have shown that Keap1–Nrf2 complex formation is a central mechanism controlling cellular antioxidant capacity through the regulation of either Nrf2 or Keap1 (refs.^30,44^). Keap1 is the primary repressor of Nrf2, whereas PGAM5, a mitochondrial membrane-associated protein and putative mitochondrial stress hub, has traditionally been regarded as a Keap1 substrate involved in mitochondrial quality control^21^. However, in our study, Keap1 knockout did not alter PGAM5 expression, suggesting that PGAM5 may have an unappreciated upstream or regulatory role in AML cells. Our data identify PGAM5 as an essential mitochondrial retrograde signalling hub that links stromal P2RX7 activation to Nrf2-driven chemoresistance, a mechanism that has not previously been described in AML or, to our knowledge, in other cancers. The current evidence supports a relay model in which PGAM5 engages Keap1, coinciding with reduced Keap1–Nrf2 binding and decreased Nrf2 ubiquitination. Notably, under basal monoculture conditions, PGAM5 deficiency had little effect on Nrf2 protein levels. By contrast, after co-culture with stromal cells, PGAM5 knockout significantly reduced Nrf2 protein abundance in AML cells. Pharmacological modulation of mtROS using the mitochondria-targeted oxidant mitoPQ or antioxidant mitoTEMPO further showed that PGAM5-knockout cells failed to transmit mitochondrial stress signals to Nrf2. These results indicate that PGAM5 functions in a stress-dependent manner, consistent with previous reports^18,19^. Post-translational modifications of Keap1, including succinylation^45^, S-lactation^46^ and phosphorylation^47^, have been shown to promote Keap1 dissociation from Nrf2 and thereby stabilize Nrf2. In addition, phosphorylation of Nrf2 at Ser40 within the Neh2 domain is a key activating modification that releases Nrf2 from Keap1 and inhibits ubiquitin-proteasomal degradation^48,49^. Given the emerging role of PGAM5 in regulating post-translational signalling events, whether PGAM5 directly contributes to these Keap1 or Nrf2 modifications remains an important question for future investigation.

Accumulating evidence indicates that AML chemoresistance is driven by the coordinated activation of multiple intrinsic and microenvironment-dependent pathways rather than by a single mechanism. For example, BMSC-derived MMP14 promotes AML progression and chemoresistance through activation of JAK–STAT signalling^40^. IL-33-dependent NFκB activation inhibits apoptosis, enhances proliferation and reduces chemosensitivity, whereas these effects can be reversed by the NFκB inhibitor Bay-117085 (ref.^50^). In addition, activation of the RAS/MAPK pathway enables AML cells to evade Bcl-2 inhibitors by increasing Mcl-1 protein stability^51^. These studies underscore the multifactorial nature of AML drug resistance, involving not only antioxidant adaptation but also cytokine signalling, apoptotic rewiring and oncogenic kinase activation. Although P2RX7-dependent Ca^2+^ signalling has been implicated in solid tumour through pathways such as PI3K-AKT and ERK1/2 (ref.^39^), the role of P2RX7-mediated Ca^2+^ influx in AML chemoresistance has remained incompletely defined. Our work positions mitochondrial redox stress as a complementary mechanism to well-established drivers of chemoresistance in AML. A follow-up study with a larger, genotype-stratified primary AML cohorts and patient-derived xenograft models will be needed to determine whether this axis is preferentially active in specific molecular subtypes and whether it functionally intersects with other resistance pathways, such as PI3K/AKT signalling or Bcl-2 family-mediated survival. These investigations may help to define patient subsets in which the P2RX7–PGAM5–Nrf2 pathway may constitute a targetable vulnerability for combination therapy.

In summary, our study reveals that P2RX7-triggered mitochondrial stress enhances the antioxidant capacity of AML cells through PGAM5-mediated activation of the Nrf2/ARE signalling pathway. In this process, PGAM5 serves as a critical effector that couples mitochondrial retrograde signalling to nuclear antioxidant responses. These findings provide mechanistic insight into how the BMME contributes to chemoresistance and identify P2RX7 as a promising therapeutic target.

## Supplementary information

Supplementary information

## Data Availability

Data files will be made available upon reasonable request to the corresponding authors.
