# Supplementary material for: Bone marrow stromal cells promote chemoresistance in acute myeloid leukaemia through P2RX7-mediated antioxidant defence
Source: Exp Mol Med. 2026 Sep 3;58(9):3025–41. doi: 10.1038/s12276-026-01839-4 (PMC13633263; doi:10.1038/s12276-026-01839-4)
Supplement: Supplementary file 1 [file 12276_2026_1839_MOESM1_ESM.pdf]

Supplementary Figure Legends

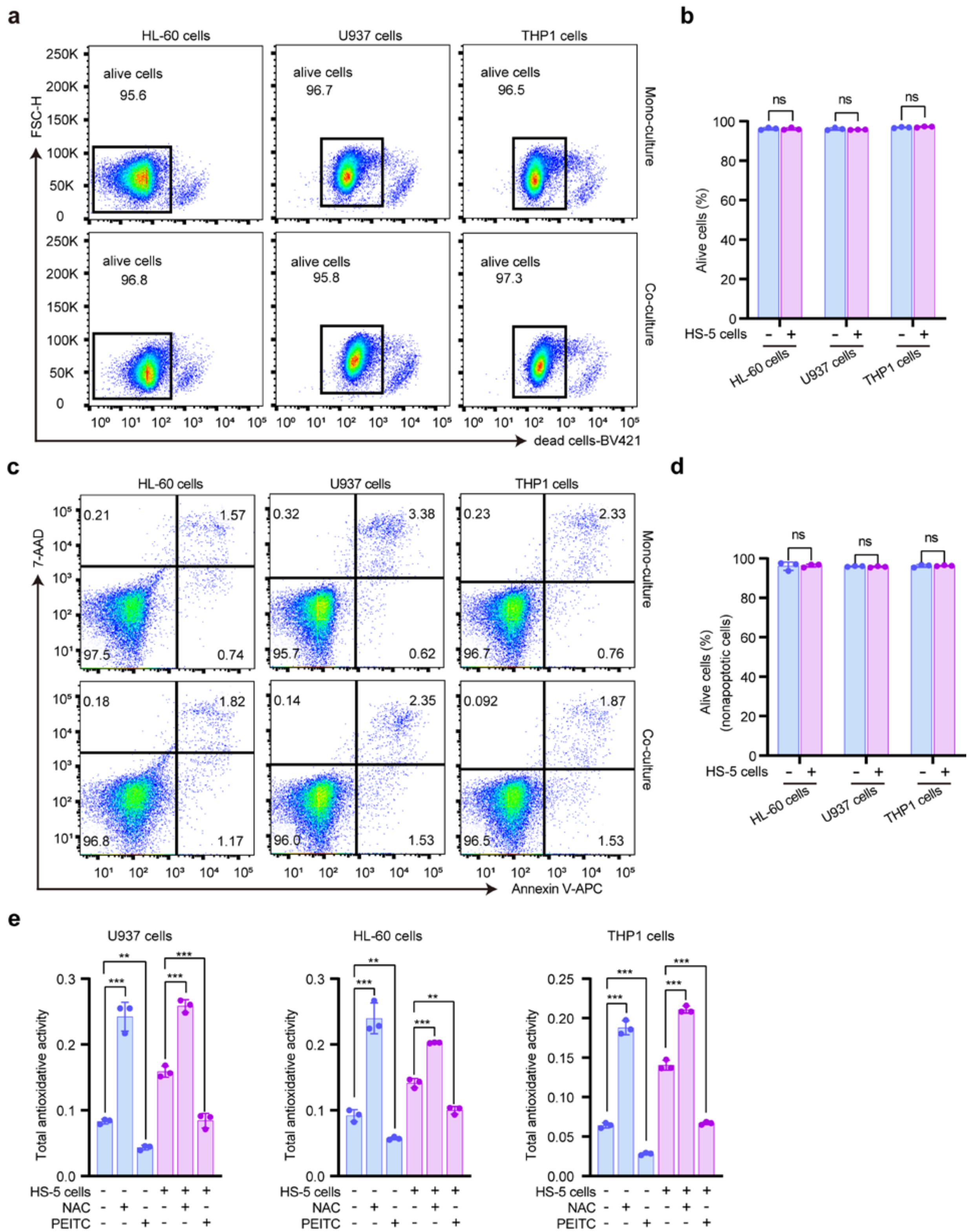

Supplementary Fig. 1. BMSCs confer oxidative stress tolerance without compromising AML cell viability (related to Fig. 1). (a) Representative flow cytometry (FCM) profiles of viable and dead AML cells labeled with fixable viability dye. (b) Quantification of live cell percentages from (a) (n = 3 independent experiments). (c) FCM profiles of apoptosis in AML cells co-cultured with or without HS-5 cells. (d) Quantification of live cells from (c) (n = 3 independent experiments). (e) Total antioxidant activity in AML cells treated with NAC (1 mM, 1 h) or PEITC (5 μM, 5 h), measured by ABTS assay (n = 3 independent experiments). Data are mean ± SD. *$P < 0.05$, **$P < 0.01$, ***$P < 0.001$, ns: not significant.

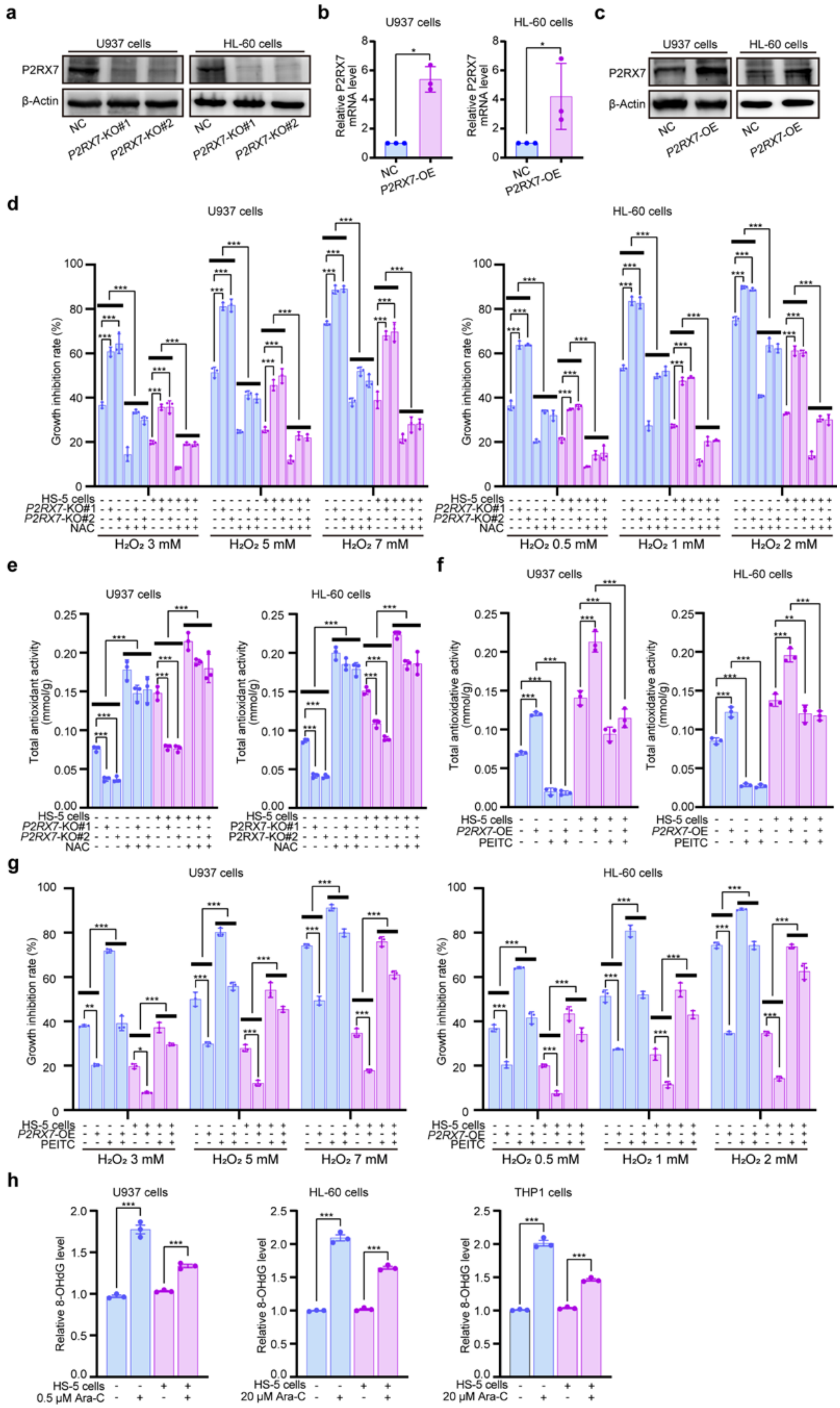

a
U937 cells
HL-60 cells
P2RX7
β-Actin
NC
P2RX7-KO#1
P2RX7-KO#2
b
U937 cells
HL-60 cells
Relative P2RX7 mRNA level
NC
P2RX7-OE
c
U937 cells
HL-60 cells
P2RX7
β-Actin
NC
P2RX7-OE
d
U937 cells
HL-60 cells
Growth inhibition rate (%)
HS-5 cells
P2RX7-KO#1
P2RX7-KO#2
NAC
H2O2 3 mM
H2O2 5 mM
H2O2 7 mM
H2O2 0.5 mM
H2O2 1 mM
H2O2 2 mM
e
U937 cells
HL-60 cells
Total antioxidant activity (mmol/g)
HS-5 cells
P2RX7-KO#1
P2RX7-KO#2
NAC
f
U937 cells
HL-60 cells
Total antioxidative activity (mmol/g)
HS-5 cells
P2RX7-OE
PEITC
g
U937 cells
HL-60 cells
Growth inhibition rate (%)
HS-5 cells
P2RX7-OE
PEITC
H2O2 3 mM
H2O2 5 mM
H2O2 7 mM
H2O2 0.5 mM
H2O2 1 mM
H2O2 2 mM
h
U937 cells
HL-60 cells
THP1 cells
Relative 8-OHdG level
HS-5 cells
0.5 μM Ara-C
20 μM Ara-C

Supplementary Fig. 2. P2RX7 modulates redox adaptation and DNA damage resistance in AML cells (related to Fig. 2). (a) Western blot confirming P2RX7 knockout efficiency (n = 3 independent experiments). (b-c) RT-qPCR (b) and western blot (c) confirming P2RX7 overexpression (n = 3 independent experiments). (d-e) H_2O_2 tolerance (d) and total antioxidant activity (e) in P2RX7-knockout AML cells treated with NAC (1 mM, 1 h) (n = 3). (f-g) Antioxidant activity (f) and H_2O_2 tolerance (g) in P2RX7-overexpressing AML cells treated with PEITC (5 μM, 5 h) (n = 3 independent experiments). (h) 8-OHdG levels in AML cells following 24 h Ara-C treatment, measured by ELISA (n = 3 replicates). Data are mean ± SD (b, e-g) or mean ± SEM (h). *P < 0.05, **P < 0.01, ***P < 0.001.

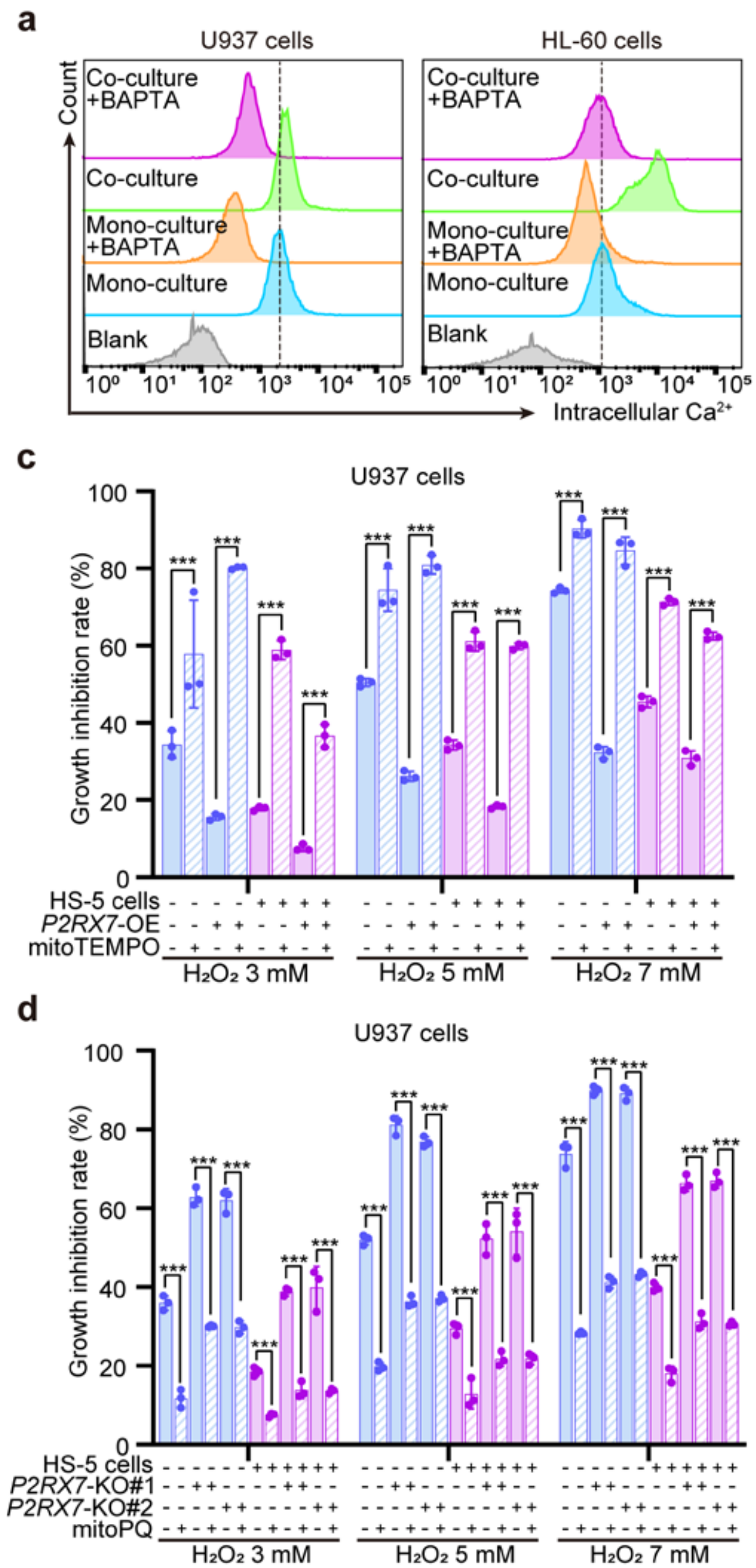

Supplementary Fig. 3. P2RX7 modulates oxidative stress via calcium signaling and mitochondrial ROS (related to Fig. 3). (a) FCM profiles of intracellular Ca^{2+} in AML cells treated with BAPTA (20 μM, 6 h), indicated by Calbryte 630 AM fluorescence. (b) Quantification of intracellular Ca^{2+} levels in AML cells from (a) (n = 3 independent

experiments). MFI, median fluorescence intensity. (c) H_2O_2 tolerance in P2RX7-overexpressing AML cells treated with mitoTEMPO (100 μM, 1 h) (n = 3 independent experiments). (d) H_2O_2 tolerance in P2RX7-knockout AML cells treated with mitoPQ (20 μM, 1 h) (n = 3 independent experiments). Data are mean ± SD. *P < 0.05, **P < 0.01, ***P < 0.001.

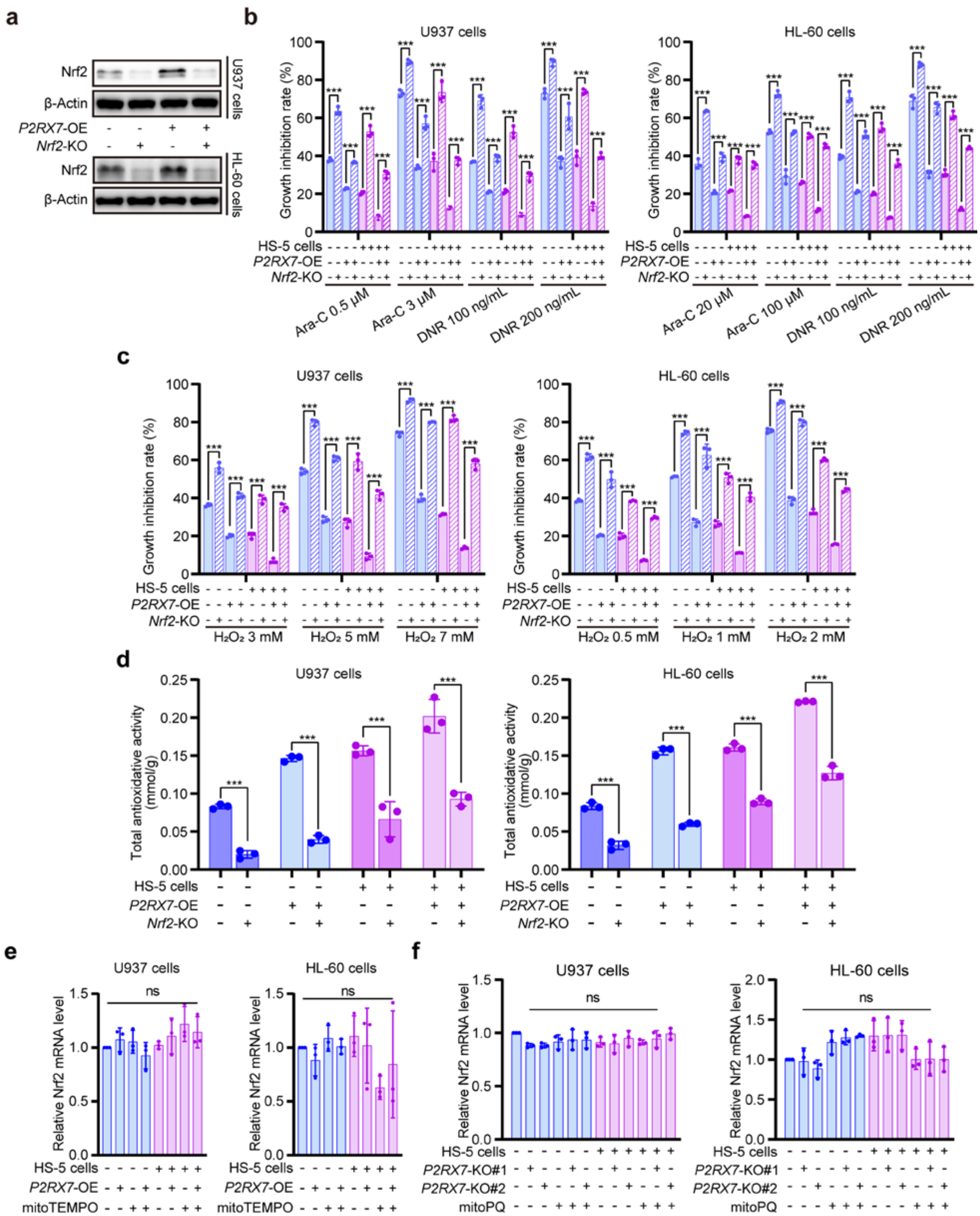

Supplementary Fig. 4. Nrf2 is required for P2RX7-mediated antioxidant programming in AML (related to Fig. 4). (a) Western blot confirming Nrf2 knockout

efficiency in P2RX7-overexpressing AML cells (n = 3 independent experiments). (b-d) Chemosensitivity (b), H_2O_2 tolerance (c), and antioxidant activity (d) of P2RX7-overexpressing AML cells with or without Nrf2 knockout (n = 3 independent experiments). (e) Nrf2 mRNA levels in P2RX7-overexpressing AML cells treated with mitoTEMPO (100 μM, 1 h), assessed by RT-qPCR (n = 3 independent experiments). (f) Nrf2 mRNA levels in P2RX7-knockout AML cells treated with mitoPQ (20 μM, 1 h) (n = 3 independent experiments). Data are mean ± SD. *P < 0.05, **P < 0.01, ***P < 0.001, ns: not significant.

a

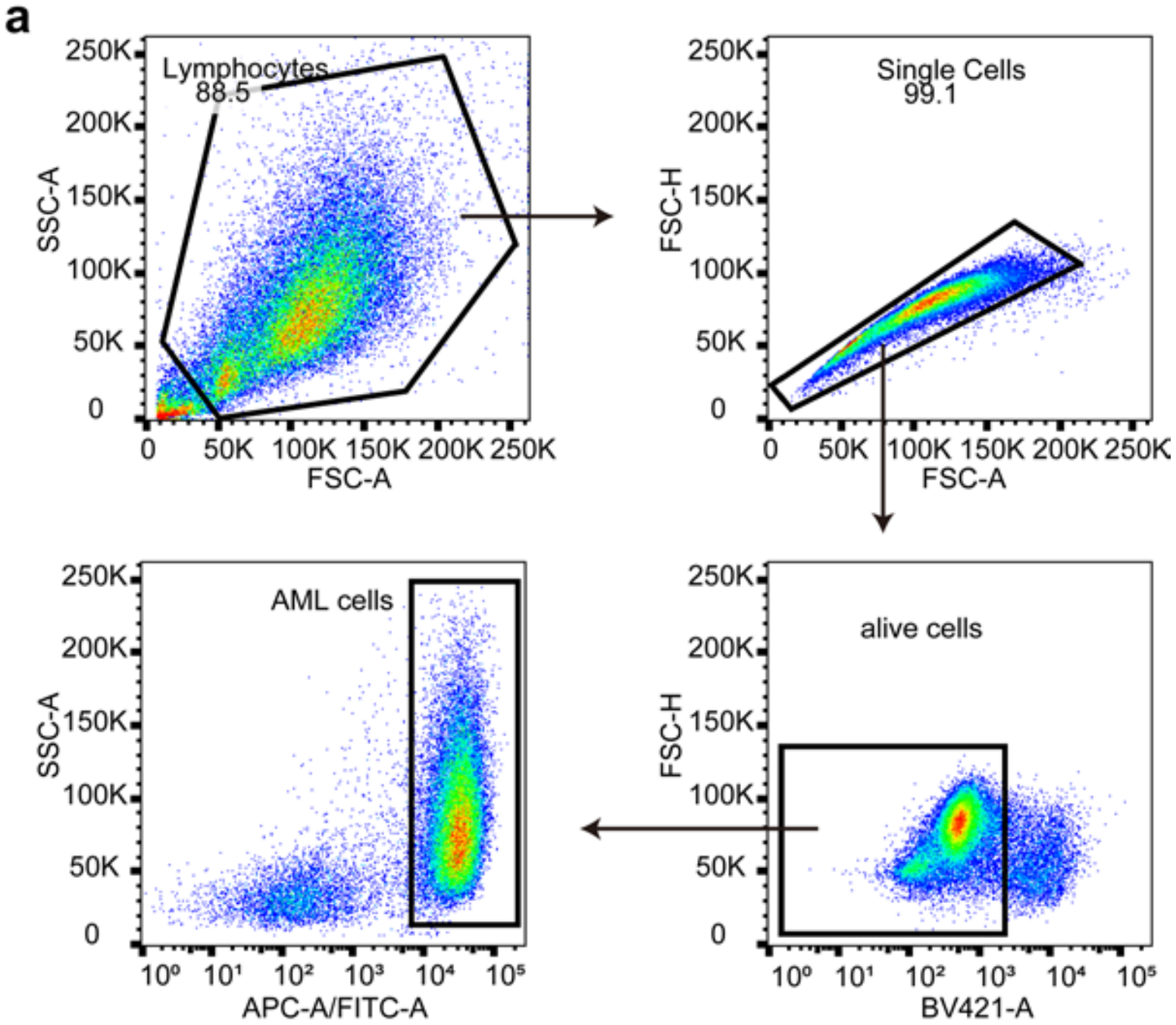

Supplementary Fig. 5. Flow-cytometry gating hierarchy for leukemic burden quantification (related to Fig. 6).
